# Exploratory study of antibody titers against SARS-CoV-2 using an indirect immunoperoxidase assay in COVID-19 patients and vaccinated volunteers

**DOI:** 10.1186/s41182-024-00635-y

**Published:** 2024-09-29

**Authors:** Shungo Katoh, Ikkoh Yasuda, Kazuhiro Kitakawa, Sugihiro Hamaguchi, Eiichiro Sando

**Affiliations:** 1 Department of General Internal Medicine and Clinical Infectious Diseases, Fukushima Medical University, Fukushima, Japan; 2 Department of General Internal Medicine and Infectious Diseases, Kita-Fukushima Medical Center, Fukushima, Japan; 3 Institute of Rickettsioses, Kita-Fukushima Medical Center, Fukushima, Japan; 4 Imadachi Internal Medicine Clinic, Fukuoka, Japan; 5 Department of Microbiology, Fukushima Prefectural Institute of Public Health, Fukushima, Japan; 6 Department of General Internal Medicine, Fukushima Medical University Hospital, Fukushima, Japan

**Keywords:** COVID-19, Antibody test, Indirect immunoperoxidase assay, ELISA, ROC analysis, Accuracy

## Abstract

**Background:**

A number of antibody test kits for detecting prior SARS-CoV-2 infection and post-immunization status have been commercialized. Indirect immunoperoxidase assay (IIP) is a conventional method to test antibodies. We evaluated the diagnostic accuracy and antibody titer profile of the IIP in COVID-19 and pre- and post-vaccination.

**Methods:**

We conducted a hospital-based observational study in Fukushima prefecture, Japan. We enrolled COVID-19 inpatients who tested positive by PCR. We used serum samples collected > 10 years before the pandemic as the negative control. We also included volunteers vaccinated at the hospital. All participants were tested using an IIP with whole-cell antigen of the six SARS-CoV-2 variants isolated in Japan during the epidemic and an IgG ELISA kit. Negative controls and vaccinated volunteers were also tested using a lateral flow assay (LFA) kit. We conducted receiver operating characteristic (ROC) analysis to evaluate diagnostic accuracy and performed logistic regression analysis to explore factors associated with antibody titer.

**Results:**

We included 146 COVID-19 inpatients, 38 negative controls, and 36 vaccinated volunteers. Most participants had the highest titer for IgG and IgM in the wild type-A antigen among the six variants. The sensitivity, specificity, and accuracy of the IgG ELISA kit were 60.3%, 100%, and 68.5%; of the IIP for IgG with the cutoff titer at 1:80, 82.2%, 94.7%, and 84.8%, respectively. The ROC curves of the ELISA and IIP for IgG were almost identical. In the IgG tests of the 36 volunteers, 35 were positive for ELISA and IIP and 34 for LFA after two vaccinations. IgM titers in the IIP were <  = 1:40 in 114 patients and 32 volunteers after two vaccinations; therefore, the IgM titer is unsuitable for diagnosis. In COVID-19 patients, age, days from disease onset, >  = 7 days after the second vaccination, and immunosuppressants for comorbidity were associated with IgG titer of >  = 1:640 in the IIP.

**Conclusions:**

The diagnostic accuracy of the IIP for detecting IgG antibodies in COVID-19 or after two vaccinations is equivalent to that of an ELISA. Further investigations are required to address the association between antibody titers in the IIP and their protective or harmful effects against COVID-19.

**Supplementary Information:**

The online version contains supplementary material available at 10.1186/s41182-024-00635-y.

## Introduction

COVID-19, an infectious disease caused by a novel coronavirus known as SARS-CoV-2, was first reported in Wuhan, China, in December 2019. Since then, it has become a global epidemic, with escalating outbreak numbers [1]. Despite the development of vaccination efforts to prevent and control severe cases of the disease, the virus continues to mutate, and the COVID-19 epidemic is anticipated to persist in the future. While an increasing number of individuals have developed immunity to the virus through either natural infection or vaccination, it is not recommended to use antibody testing for the diagnosis or treatment of COVID-19 [2, 3]. Nonetheless, antibody testing can be employed when there is a need to confirm prior infection or vaccination against SARS-CoV-2 [3]. Several anti-SARS-CoV-2 antibody tests are commercially available; however, their diagnostic accuracy varies, and some tests may be less sensitive than other semi-automated assays, such as enzyme-linked immunosorbent assay (ELISA) or chemiluminescent immunoassay (CLIA) [4].

Indirect immunoperoxidase assay (IIP) and immunofluorescent assay (IFA) are considered standard serologic assays used to measure antibody titers in various diseases, including rickettsiosis [5]. In situations such as scrub typhus caused by *Orientia tsutsugamushi*, where the major antigen is highly diverse, it has been suggested that a whole-cell antigen test utilizing locally prevalent strains may provide superior diagnostic accuracy compared to a test kit using a recombinant protein as the antigen [6]. Similarly, in the case of SARS-CoV-2, where new mutant variants are causing epidemics in succession, there is a possibility that a recombinant protein-based test kit may have different diagnostic accuracy from an antibody test that uses a whole-cell antigen.

The present study aims to compare the diagnostic accuracy of the IIP for antibody testing with several variants of the SARS-CoV-2 isolated in Japan. The comparison will be made with a commercially available ELISA kit for both SARS-CoV-2 infected and uninfected subjects. Furthermore, the study will investigate the trends in antibody titers by IIP before and after vaccination compared to ELISA and a rapid diagnostic test (RDT) kit using lateral flow assay (LFA). Lastly, the study will explore factors associated with high antibody titers in COVID-19 patients. The results of this research are expected to provide valuable insights into the applicability of IIP to SARS-CoV-2 infection and immunization, as well as a better understanding of antibody titers in COVID-19.

## Methods

### Study design, participants, and enrollment criteria

We conducted an observational study at Kita-Fukushima Medical Center, a medical institution located in Fukushima Prefecture in Japan that had up to 20 specially designated beds for COVID-19 patients and was considered a priority institution for COVID-19 cases. The hospital received COVID-19 patients requiring hospitalization, mainly from northern Fukushima Prefecture, as well as some from other regions of the prefecture. Notably, the medical center does not possess an intensive care unit for COVID-19; therefore, severe cases were preferentially referred to other tertiary care hospitals. The study's primary objective, the investigation of the diagnostic accuracy of the IIP for antibody testing with several variants of the SARS-CoV-2 isolated in Japan, was designed as a two-gate case–control diagnostic accuracy study. Inclusion criteria for the cases were hospitalized patients with SARS-CoV-2 infection confirmed by reverse transcription polymerase chain reaction (RT-PCR) and whose consent was obtained by written signature by the patient or a surrogate decision-maker; children younger than 15 years and cases diagnosed by methods other than RT-PCR were excluded. The inclusion period for the study was approximately one year, from February 2021 to February 2022. During this period, participants were prospectively included from July 2021 to February 2022. Retrospective inclusion was also available for COVID-19 patients admitted between February and June 2021 who had provided consent for laboratory specimen storage and had not indicated any intention to opt out of participating in the study. The negative control group comprised serum samples sent from various locations across Japan for rickettsioses and tularemia diagnostic testing from October 2006 through July 2008, all of which tested negative in the prior tests. Negative control samples were serum specimens collected more than a decade before the onset of the COVID-19 pandemic and subsequently stored at − 80 °C so that personal information could not be identified. In addition, of those scheduled for vaccination at Kita-Fukushima Medical Center from September to October 2021, adult volunteers who gave written consent to participate in the study were selected as subjects for pre- and post-vaccination antibody titer measurement.

### Study procedures and sample collection

Basic information, medical history, history of vaccination against SARS-CoV-2, presence of COVID-19 severity risk factors, severity, COVID-19-associated pneumonia, outcome, and blood test results at admission were extracted from the medical records of patients whose consent to participate in the study was obtained by the collaborating physicians. The database was anonymized by filling out a patient information form. Of the clinical laboratory specimens from hospitalized patients, serum was stored at − 80 °C and used for antibody testing. The last stored serum collected for patients with multiple blood draws performed during hospitalization was used for antibody testing. Research collaborating physicians collected blood from the fingertips of the study participants before and after vaccination using a safety lancet. The blood was collected in a filtered blood collection tube, and the serum was separated by centrifugation at approximately 2000 G for 3 min. The serum was then stored at − 80 °C. To assess the presence or absence of antibodies before and after the initial and secondary vaccination, the serum samples were collected three times: on the day of the first and second vaccination and 7–14 days after the second vaccination. These time points correspond to pre-vaccination, post-first, and post-second vaccination, respectively.

### Indirect immunoperoxidase assay

The antigen cells used for IIP were VeroE6/TMPRSS2 cells (ID: JCRB1819) infected with six variants provided by the National Institute of Infectious Diseases, Japan (Additional file 1). The antigen cells were prepared in the biosafety level 3 laboratory of the Fukushima Prefectural Institute of Public Health, inactivated in a 4% paraformaldehyde phosphate-buffered solution for 30 min, and then suspended in 1% fetal bovine serum + 0.1% formaldehyde added phosphate-buffered saline (PBS). The suspensions were transported to the Institute of Rickettsioses at Kita-Fukushima Medical Center while refrigerated in a cooler box and then stored at − 80 °C. Slide preparation and staining for IIP were performed as outlined in the IIP for rickettsiosis by Suto and Fujita [7, 8]. However, the test sera were treated by absorption with a negative control cell suspension uninfected with SARS-CoV-2 to exclude nonspecific binding of antibodies to the antigen cells (Additional file 1). Briefly, six variant antigen cells (wild type-A, alpha, delta, omicron BA.1.1, omicron BA.2, omicron BA.5) and negative control cell suspension were spotted on a glass slide, air dried at 37 °C for 30 min, then fixed it in acetone at − 20 °C for 10 min under light-shielded conditions. After drying, the slide-antigen was used immediately, otherwise stored at − 20 °C. After the twice absorption process, patient sera were diluted twofold from 1:40 to 1:10,240 with PBS containing 0.3% bovine serum albumin, and 0.01 mL of each dilution was applied to the spot of the antigen on the slide. The slide was incubated for 30 min at 37 °C in a humidified chamber, and washed twice for 5 min in PBS. Then, 0.01 mL of 1:100-diluted antihuman IgG or IgM rabbit serum (Dako Agilent Technologies Japan, Tokyo, Japan) was added to each spot, followed by an incubation for 30 min at 37 °C in the chamber and washed as described above. The slide was finally incubated in the chamber filled with freshly prepared enzyme substrate solution composed of 1 volume of 80% ethanol containing 0.2% 4-Cl-1-naphtol, 4 volumes of PBS, and 0.01 volume of 3% hydrogen peroxide at room temperature for 5 min under light-shielded conditions. This was washed three times by changing the solution to distilled water in the chamber and air dried. The slide was then covered with glycerol gelatin and a coverslip. The results were read visually through a microscope at a power of × 100 – × 400. The titer was determined as the highest dilution of the serum, which demonstrated blue or blue black-colored cellular surface dots (Additional file 2).

### Enzyme-linked immunosorbent assay

The commercially available IgG ELISA kit, Anti-SARS-CoV-2 ELISA (EUROIMMUN Japan, Tokyo, Japan), was used as the antibody test for comparison. According to the manufacturer’s instruction, the ELISA kit used 12 microplate strips, each containing eight individual break-off wells in a frame coated with the antigen, which consisted of recombinant S1-domain of the spike protein of SARS-CoV-2 in the human cell line HEK 293. The sera were diluted at 1:101 with the ready-for-use sample buffer, which was tested with positive control, negative control, and a calibrator containing human IgG within the same microplate flame. The test kit also contains enzyme conjugate solution, substrate solution, stop solution, and wash buffer. Following the manufacturer’s instructions, we manually washed the microplate wells three times using 300 μL of working-strength wash buffer by a multi-channel pipette for each washing process. The absorbance at 450 nm was measured as optical density (OD) with Microplate Reader MPR-A100T (AS ONE Corporation, Osaka, Japan). The OD ratio, which is the OD value divided by the calibrator’s OD, was calculated to minimize inter-assay variation, and those with an OD ratio > 1.1 were judged as a positive result following the manufacturer’s recommendation.

### Lateral flow assay

Samples from vaccinated volunteers and negative controls were also tested with a commercially available LFA kit, KBM COVID-19 IgG/IgM (KOHJIN BIO, Saitama, Japan). According to the manufacturer’s instructions, the kit can test 10 μL of serum or plasma or 20 μL of whole blood, which are applied to the specimen well with two drops (approximately 80 μL) of sample buffer. The kit is a kind of RDT using the spike protein of SARS-CoV-2 as an antigen. After a 15-min reaction time, visually check for the appearance of IgG and IgM lines; if control lines do not appear, the test is considered invalid. We used sera for the LFA accordingly. In the rare case of insufficient sample volume, we diluted the serum with PBS to 10 μL.

### Sample size estimation and statistical analysis

To determine the required sample size for our study, we utilized the sample size calculator of easyROC [9]. Our study involves the comparison of two diagnostic tests: the Anti-SARS-CoV-2 ELISA kit for IgG, with a reported area under the receiver operating characteristic (ROC) curve (AUC) of 0.99 [10], and the IIP measuring IgG antibody titer. We set the null hypothesis for the IIP at an AUC of 0.99, with the alternative hypothesis suggesting that the IIP has an AUC of less than 0.90, indicating a difference greater than 0.09. To achieve 90% power with a significance level of 0.025 on each side and a 1:1 case–control ratio, we calculated that 35 cases in each group are needed. Due to the potential for laboratory errors, we aimed for 38 cases in both the vaccination and negative control groups. We aimed to measure antibody titers in as many consenting COVID-19 patients as possible, considering potential variations in SARS-CoV-2 variants during the study period, to explore potential differences in test results due to variant variations.

The study utilized ROC analysis to assess the accuracy of different antibody tests and establish cutoff values for the IIP test. Categorical variables were presented as frequencies and percentages, while continuous variables were summarized as mean or median, alongside standard deviation or interquartile range. The antibody titer of IIP was determined as the reciprocal of the dilution factor, with 1 designated for < 40 and 10,240 for > 10,240. Antibody titers were analyzed as continuous variables, and natural log transformations were applied as necessary. Furthermore, odds ratios with 95% confidence intervals were computed using logistic regression analysis with multiple imputations to handle missing data. Missing values were imputed using chained equation models encompassing all variables with missing values and those potentially correlated with them or the outcome variable. The analysis used STATA, version 15.1 (StataCorp LLC, College Station, TX). All tests were two-tailed, with a significance level set at *P* < 0.05.

### Ethics statement

Written, informed, and signed consent was obtained from all adult vaccination volunteers and COVID-19 patients or guardians. Blood sampling for research was omitted to minimize invasive interventions in hospitalized patients, as serum stored in the clinical laboratory was utilized. Data entry and analysis were carried out anonymously. Serum samples stored between 2006 and 2008 were used as negative controls. Individual consent was not obtained because the personal information was anonymized, and individual identification was impossible. This research was designed in 2021 in compliance with the Ethical Guidelines for Medical and Health Research Involving Human Subjects in Japan. This study was approved by the institutional review boards and independent ethics committees of Kita-Fukushima Medical Center (approval number: 97-4) and Fukushima Medical University (approval number: 2021-153).

## Results

### Investigation flow

**Fig. 1:**
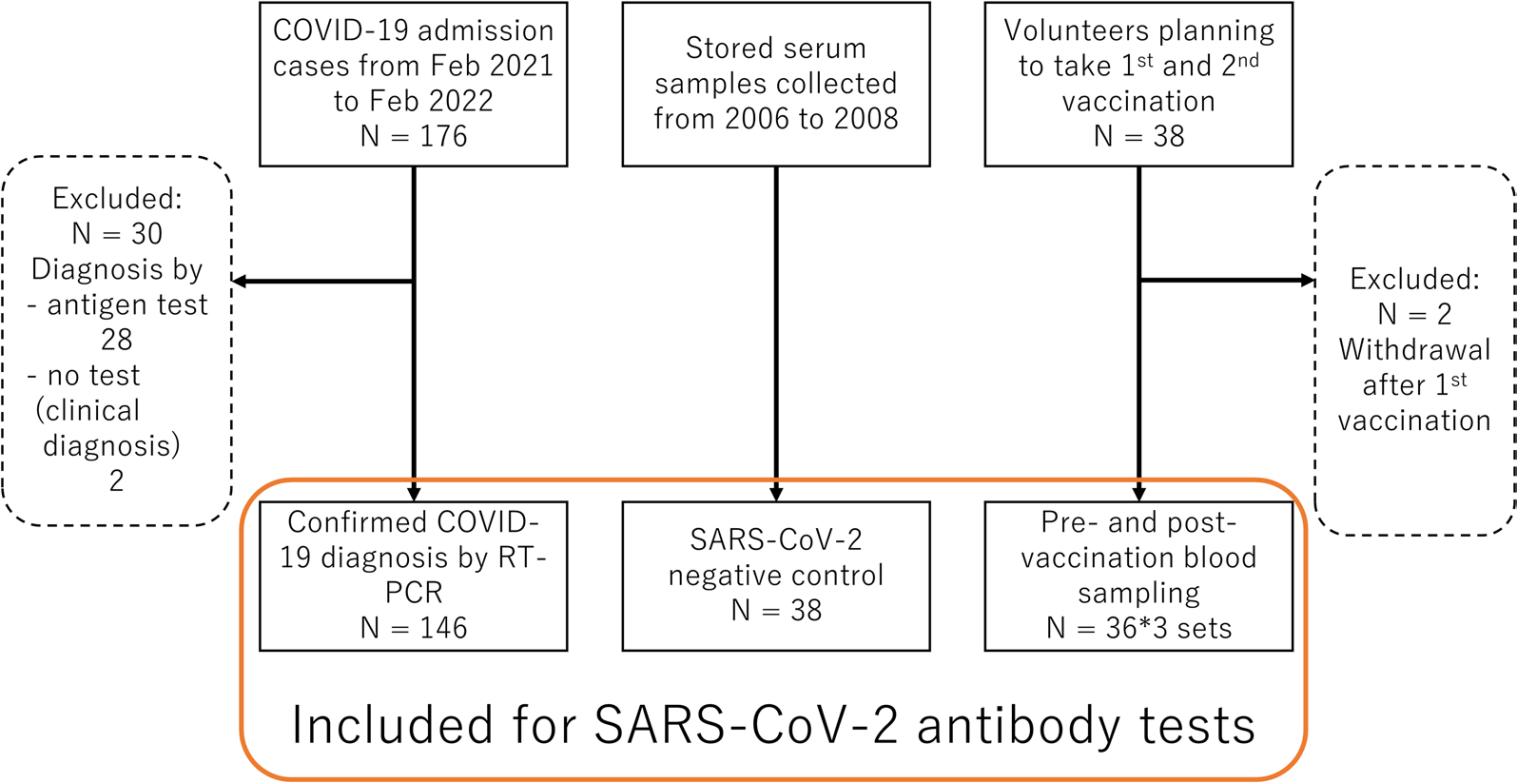
Investigation flow of the study. Samples from all participants were tested by indirect immunoperoxidase assay and enzyme-linked immunosorbent assay. In addition, pre- and post-vaccination samples and negative control samples were tested by lateral flow assay

During the study period, consent was obtained from 176 COVID-19 hospitalized patients. Thirty patients were excluded because they had been diagnosed by methods other than RT-PCR, leaving 146 patients recruited for the study. Additionally, 38 volunteers scheduled for vaccination were recruited, with 36 patients ultimately participating after two declined following the first vaccination. The study also used 38 stored negative control specimens as planned (Fig. 1).

### Status of all antibody tests

All serum samples were tested by ELISA and IIP as planned. Among the pre-vaccination samples, due to insufficient sample volume, one volunteer (ID: 80) underwent LFA testing with a twofold dilution, while another (ID: 107) underwent testing with a tenfold dilution. A third volunteer (ID: 112) was unable to undergo LFA testing. After the first vaccination, two volunteers (ID: 86 and 104) were tested for LFA with a twofold dilution due to insufficient serum volume. All serum samples after the second vaccination were tested for LFA as planned.

In the IIP testing of six variants, it was observed that the IgG antibody titers against the wild type-A (WT-A) were predominant in all specimens except for one hospitalized patient (ID: 74). Patient 74 displayed the highest IgG titer against the delta variant at 320, followed by WT-A and the alpha variant at 160. Similarly, the IgM antibody titers against WT-A were dominant in all samples except one hospitalized patient (ID: 52). This patient exhibited the highest IgM titer against the alpha variant and the omicron variant BA.5 at 80. In contrast, all other variants were negative at < 40. As a result, the highest IIP antibody titer among the six variants was used as the representative value in subsequent analyses.

### Results of IIP and ELISA among COVID-19 patients

**Fig. 2:**
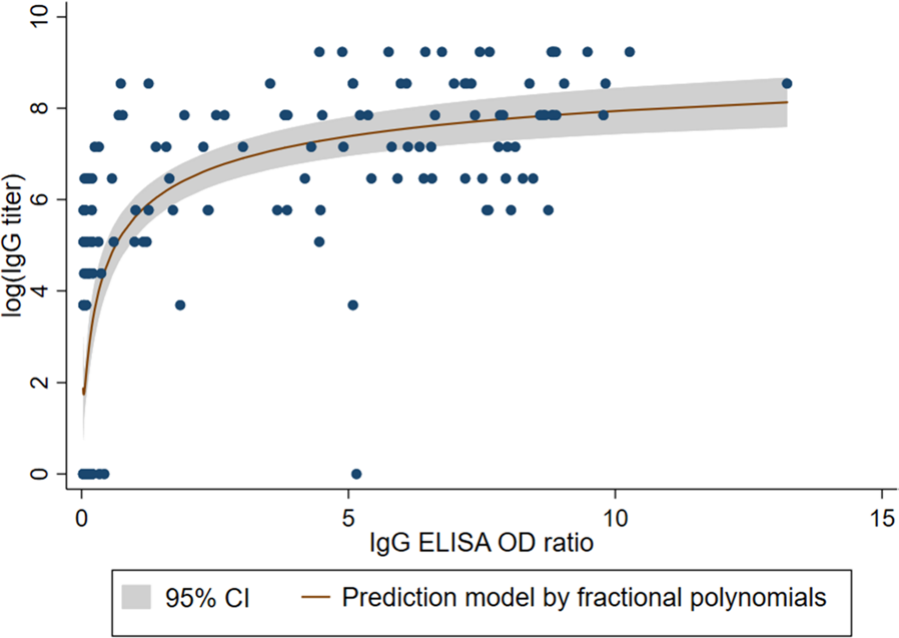
IgG ELISA OD ratio and log (IgG titer) by IIP among COVID-19 patients and controls. The scatter plot displays optical density ratios by enzyme-linked immunosorbent assay for IgG and the natural logarithm of IgG titers by indirect immunoperoxidase assay among COVID-19 patients and controls. A fitted curve prediction model with fractional polynomials and a 95% confidence interval range are also depicted. The estimated Spearman’s rank correlation coefficient is 0.778 (95% confidence interval: 0.714–0.830), which indicates a strong correlation

In COVID-19 inpatients, there was a strong correlation between the natural logarithm of IgG antibody titer measured by IIP and the OD ratio measured by ELISA (Spearman’s rank correlation: 0.778, 95% CI: 0.714–0.83) (Fig. 2). Among 146 inpatients, 117 had a known number of days from disease onset to blood collection. It was observed that the OD ratio and IgG antibody titer in both ELISA and IIP increased with the number of days passed. Additionally, the IgM antibody titer measured by IIP was negative at < 40 in 103 (70.5%) of 146 hospitalized patients, and no clear correlation was found between the number of days elapsed from disease onset to blood collection and IgM antibody titer (Additional file 3).

**Fig. 3:**
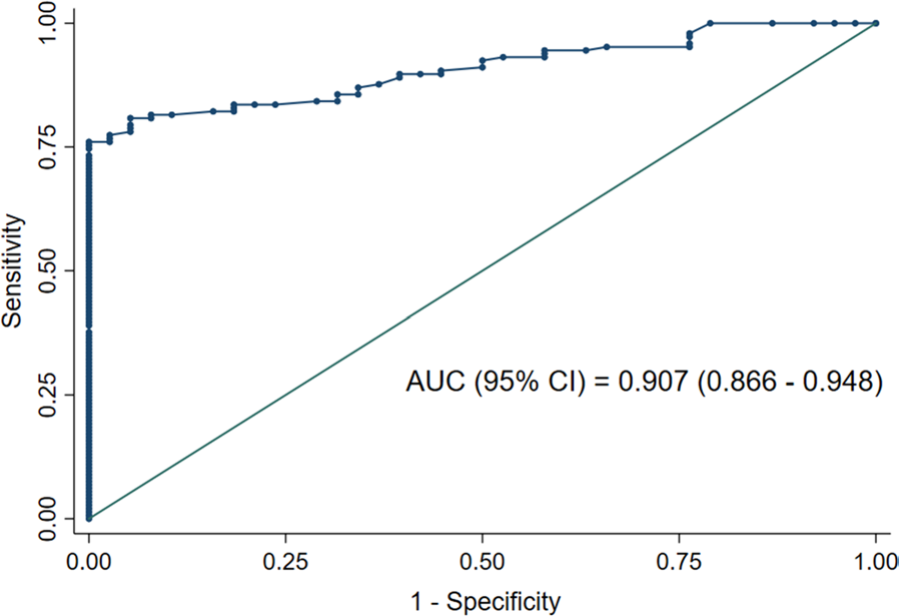
ROC curve of IgG ELISA. The graph displays a receiver operator characteristic curve for the IgG test by enzyme-linked immunosorbent assay for COVID-19. The estimated area under the curve is 0.907 (95% confidence interval: 0.866–0.948)

**Fig. 4:**
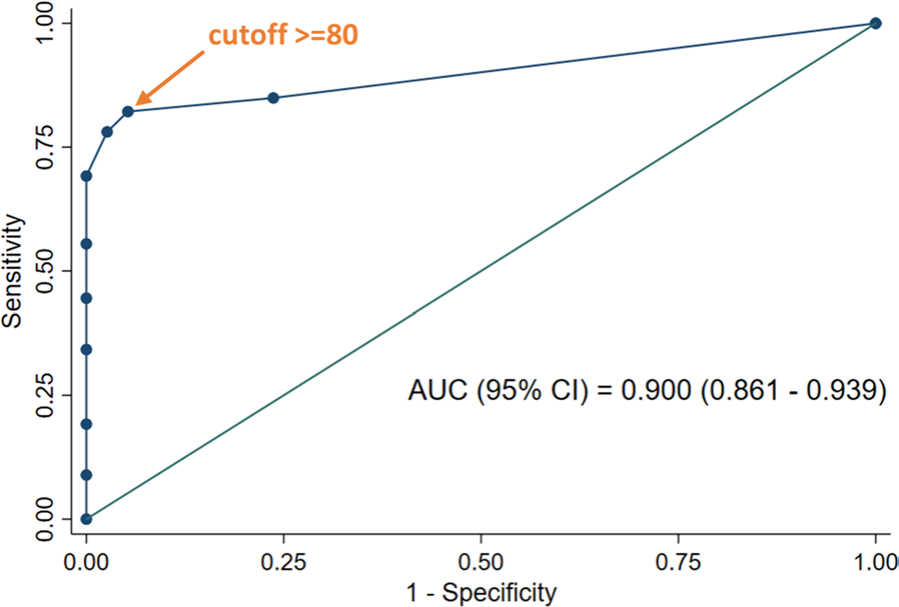
ROC curve of IgG test by IIP. The graph displays a receiver operator characteristic curve for the IgG test by indirect immunoperoxidase assay for COVID-19. The estimated area under the curve is 0.900 (95% confidence interval: 0.861–0.939). The arrow signifies the plot of the cutoff titer at 80, which is closest to the upper left corner

Among the COVID-19 patients, 88 tested positive, and 58 tested negative using ELISA. The ROC analysis for the diagnostic performance of ELISA showed an AUC of 0.907 (95% CI: 0.866–0.948) (Fig. 3). At a cutoff OD ratio of 1.1, the sensitivity was 60.3% (95% CI: 51.9–68.3%), the specificity was 100% (95% CI: 90.7–100%), and the accuracy was 68.5% (95% CI: 61.2–75.1%) (Table 1). Regarding the IgG test by IIP, the highest accuracy recorded was 84.8% (95% CI: 78.8–89.6%) with a cutoff titer of 80. At this cutoff, the sensitivity was 82.2% (95% CI: 75.0–88.0%), and the specificity was 94.7% (95% CI: 82.3–99.4%) (Table 1). The AUC for the IgG test by IIP was 0.900 (95% CI: 0.861–0.939), closely resembling the ROC curve for ELISA (Fig. 4).

**Table 1:** Diagnostic 2 × 2 tables and parameters of IgG test by ELISA and IIP

IgG ELISA OD ratio > 1.1	Patients	Controls	Total	Proportion (%) (95% CI)
Positive	88	0	88	PPV = 100 (95.9–100)
Negative	58	38	96	NPV = 39.6 (29.7–50.1)
Total	146	38	184	Prevalence = 79.3
Proportion (%) (95% CI)	Sn = 60.3 (51.9–68.3)	Sp = 100 (90.7–100)	Accuracy = 68.5 (61.2–75.1)	

IIP IgG titer > = 80	Patients	Controls	Total	Proportion (%) (95% CI)
Positive	120	2	122	PPV = 98.4 (94.2–99.8)
Negative	26	36	62	NPV = 58.1 (44.8–70.5)
Total	146	38	184	Prevalence = 79.3
Proportion (%) (95% CI)	Sn = 82.2 (75.0–88.0)	Sp = 94.7 (82.3–99.4)	Accuracy = 84.8 (78.8–89.6)	

*ELISA* enzyme-linked immunosorbent assay, *OD* optical density, *CI* confidence interval, *PPV* positive predictive value, *NPV* negative predictive value, *Sn* sensitivity, *Sp* specificity, *IIP* indirect immunoperoxidase assay

### Results of IIP, ELISA, and LFA among vaccinated volunteers at pre-, post-first, and post-second vaccination

**Fig. 5:**
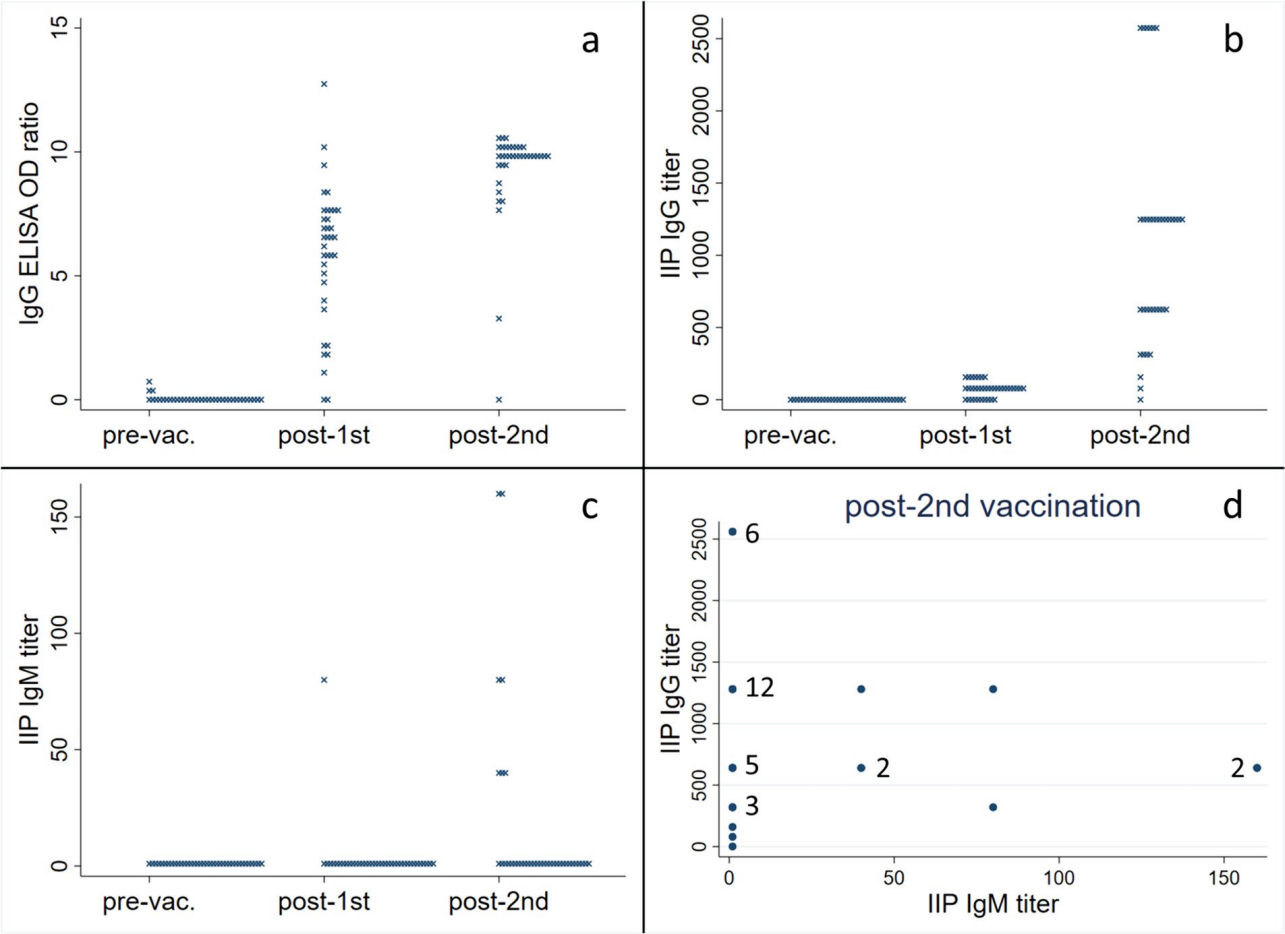
Quantitative antibody test results of the 36 vaccinated volunteers. The serum samples were collected on the day of the first (pre-vac) and second (post-1st) vaccination and 7–14 days after the second vaccination (post-2nd). **a** The dot plot displays optical density ratios by enzyme-linked immunosorbent assay for IgG before and after two vaccinations. **b** The dot plot displays the IgG titers by indirect immunoperoxidase assay before and after two vaccinations. **c** The dot plot displays the IgM titers by indirect immunoperoxidase assay before and after two vaccinations. **d** The scatter plot displays IgG and IgM titers by indirect immunoperoxidase assay after the second vaccination. The numbers next to the plots indicate the number of duplicates

Before vaccination, all participants tested negative for IIP, ELISA, and LFA. Subsequent tests after the first and second vaccinations revealed a noticeable increase in OD ratio by ELISA and IgG antibody titer by IIP over time, while there was no significant increase in IgM antibody titer by IIP for most participants (Fig. 5). When using a titer of >  = 80 by IIP as the cutoff, the results of IgG antibody tests were consistent between IIP and ELISA in 22 out of 36 participants after the first vaccination. Fourteen participants tested positive only for ELISA, but all results were consistent after the second vaccination. Following the first vaccination, the results of IgG and IgM antibody tests by IIP were negative in 15 out of 36 participants, while the remaining 21 had discordant results. Even after the second vaccination, only four out of 36 participants tested positive for both IgM and IgG, 31 were IgM-negative but IgG-positive, and one was negative for both IgM and IgG (Fig. 5, Table 2). After the first vaccination, the results of IgG antibody tests by IIP and LFA were consistent in 25 out of 36 participants. With one participant testing positive only for IIP after the second vaccination, all others were consistent. IgM antibody test results by IIP and LFA were negative in 33 out of 36 participants and showed discordance in three participants after the first vaccination. Following the second vaccination, both tests were negative in 20 participants and showed discordance in 16 participants, with no participants testing positive for both IgM antibody tests after vaccination (Table 2).

**Table 2:** Diagnostic 2 × 2 tables of IIP, ELISA, and LFA after the first and second vaccination

IIP	Post 1st vaccination			Post 2nd vaccination		
IgG titer	IgG ELISA (–)	IgG ELISA ( +)	Total	IgG ELISA (–)	IgG ELISA ( +)	Total
< = 40	2	14	16	1	0	1
> = 80	0	20	20	0	35	35
Total	2	34	36	1	35	36

IIP	Post 1st vaccination			Post 2nd vaccination		
IgG titer	IgM titer < = 40	IgM titer > = 80	Total	IgM titer < = 40	IgM titer > = 80	Total
< = 40	15	1	16	1	0	1
> = 80	20	0	20	31	4	35
Total	35	1	36	32	4	36

IIP	Post 1st vaccination			Post 2nd vaccination		
IgG titer	LFA IgG (–)	LFA IgG ( +)	Total	LFA IgG (–)	LFA IgG ( +)	Total
< = 40	8	8	16	1	0	1
> = 80	3	17*	20	1	34	35
Total	11	25	36	2	34	36

IIP	Post 1st vaccination			Post 1st vaccination		
IgM titer	LFA IgM (–)	LFA IgM ( +)	Total	LFA IgM (–)	LFA IgM ( +)	Total
< = 40	33*	2	35	20	12	32
> = 80	1	0	1	4	0	4
Total	34	2	36	24	12	36

*IIP* indirect immunoperoxidase assay, *ELISA* enzyme-linked immunosorbent assay, *LFA* lateral flow assay

*Two serum samples tested by × 2 dilution by LFA due to insufficient sample volume

When comparing the basic characteristics of the 16 individuals who tested negative and the 20 who tested positive for IgG antibodies by IIP after the first vaccination, it was found that the mean age was 48.6 in the negative group and 38.2 in the positive group. The positive group was significantly younger, with a mean difference of 10.4 years (95% CI: 1.5–19.3, *p* = 0.024). No other significant differences were observed (Additional file 4). Additionally, there was a moderate negative correlation between the natural logarithm of IgG antibody titers after the second vaccination and age (Spearman's rank correlation: − 0.483, 95% CI: − 0.7–0.183), indicating a tendency for relatively low antibody titers in individuals over 60 years old (Additional file 3).

### Factors associated with higher IgG titer among COVID-19 patients

Among the 146 hospitalized patients, it was found that those with an IgG antibody titer >  = 640 (*n* = 81) had significantly higher age, hypertension frequency, dyslipidemia frequency, and a longer duration from COVID-19 onset to blood sample collection compared to those with a titer < 640 (*n* = 65). The group with higher antibody titers also tended to have more frequent pneumonia findings on CT scans and more severe COVID-19 illness (Additional file 4).

Using the multiple imputation method, we conducted a multivariate logistic regression analysis to explore candidate factors associated with high antibody titers, adjusting for age, the number of days from COVID-19 onset to blood draw, and whether the second vaccination was administered at least seven days before the blood draw. We calculated the adjusted odds ratio (aOR) for an antibody titer >  = 640. We found that statistically significant factors were age group, use of immunosuppressive agents for comorbidities, days from COVID-19 onset to blood draw, and days from the second vaccination being >  = 7. The age group was divided into three categories: under 50 as a reference category, with an aOR of 3.53 (95% CI: 1.35–9.23, *p* = 0.01) for the 50–64 group, and an aOR of 1.55 (95% CI: 0.57–4.23, *p* = 0. 389) for those aged 65 and over. The use of immunosuppressive agents for comorbidities had an aOR of 0.01 (95% CI: < 0.001–0.30, *p* = 0.01), but it was scarce, with only one patient in the < 640 group and two in the >  = 640 group. The number of days from COVID-19 onset to blood draw had an aOR of 5.33 (95% CI: 1.54–18.44, *p* = 0.008) for 7–13 days and an aOR of 37.65 (9% CI: 6.36–222.9, *p* < 0.001) for >  = 14 days. The days from the second vaccination, which was >  = 7 days, had an aOR of 4.04 (95% CI: 1.27–12.87, *p* = 0.018) (Table 3).

**Table 3:** Logistic regression analysis for association with IgG titer >  = 640 in the IIP among 146 admission patients

Basic characteristics	IgG titer > = 640 frequency (%)	cOR	95% CI	*P* value*	aOR**	95% CI	*P* value*
Age as a continuous var. (*N* = 146)	81 (55.5)	1.03	1.01–1.05	**0.001**	1.01	0.99–1.04	0.17
Age < 50 (*N* = 57)	21 (36.8)	Ref			Ref		
Age 50–64 (*N* = 40)	28 (70.0)	4	1.69–9.49	**0.002**	3.53	1.35–9.23	**0.01**
Age > = 65 (*N* = 49)	32 (65.3)	3.23	1.45–7.16	**0.004**	1.55	0.57–4.23	0.389
Male sex (*N* = 76)	42 (55.3)	0.98	0.51–1.89	0.956	1.26	0.57–2.78	0.574
Chronic heart disease (*N* = 14)	7 (50.0)	0.78	0.26–2.36	0.665	0.71	0.16–3.13	0.647
Chronic lung disease (*N* = 5)	3 (60.0)	1.21	0.20–7.48	0.836	0.99	0.12–7.81	0.989
Autoimmune disease (*N* = 4)	2 (50.0)	0.8	0.11–5.82	0.823	0.1	0.01–1.76	0.116
Malignant disease (*N* = 6)	3 (50.0)	0.79	0.16–4.08	0.783	0.92	0.13–6.54	0.934
Chronic kidney disease (*N* = 4)	3 (75.0)	2.46	0.25–24.24	0.44	3.13	0.15–66.30	0.463
Diabetes mellitus (*N* = 33)	23 (69.7)	2.18	0.95–5.00	0.065	2.33	0.82–6.64	0.113
Hypertension (*N* = 60)	42 (70.0)	2.81	1.40–5.64	**0.004**	1.73	0.70–4.25	0.234
Dyslipidemia (*N* = 33)	25 (75.8)	3.18	1.32–7.65	**0.01**	2.39	0.85–6.69	0.098
Body mass index > = 30 (*N* = 8)	5 (62.5)	1.36	0.31–5.91	0.682	1.41	0.27–7.47	0.684
Steroid use for comorbidity (*N* = 6)	4 (66.7)	1.64	0.29–9.23	0.577	0.14	0.01–2.42	0.177
Immunosuppressive agent for comorbidity (*N* = 3)	2 (66.7)	1.62	0.14–18.28	0.696	0.01	0.0001–0.30	**0.01**
Current smoker (*N* = 28)^#^	13 (46.4)	0.67	0.29–1.54	0.348	0.68	0.26–1.80	0.438
Ex-smoker (*N* = 63)^#^	31 (49.2)	0.68	0.35–1.34	0.267	0.63	0.28–1.40	0.259
Days from COVID-19 onset to blood draw as a continuous var. (*N* = 117)^#^	58 (49.6)	1.36	1.18–1.55	** < 0.001**	1.26	1.11–1.44	**0.001**
Days from COVID-19 onset to blood draw: < 7 days (*N* = 22)	3 (13.6)	Ref			Ref		
Days from COVID-19 onset to blood draw: 7–13 days (*N* = 74)	35 (47.3)	5.68	1.55–20.86	**0.009**	5.33	1.54–18.44	**0.008**
Days from COVID-19 onset to blood draw: > = 14 days (*N* = 21)	20 (95.2)	126.67	12.10–1326.4	** < 0.001**	37.65	6.36–222.9	** < 0.001**
Days from 2nd vaccination to blood draw: > = 7 days (*N* = 23)	16 (69.6)	2.04	0.78–5.31	0.144	4.04	1.27–12.87	**0.018**
CT scan finding of COVID-19 associated pneumonia: none (*N* = 29)	12 (41.4)	Ref			Ref		
CT scan finding of COVID-19 associated pneumonia: in 1 lobe (*N* = 21)	7 (33.3)	0.71	0.22–2.28	0.564	0.84	0.19–3.81	0.822
CT scan finding of COVID-19 associated pneumonia: in > = 2 lobes (*N* = 96)	62 (64.6)	2.58	1.11–6.04	**0.028**	1.93	0.54–6.86	0.309
COVID-19 severity^##^: asymptomatic (*N* = 2)	1 (50.0)	1.75	0.10–31.96	0.706	1.22	0.03–44.36	0.912
COVID-19 severity^##^: mild (*N* = 22)	8 (36.4)	Ref			Ref		
COVID-19 severity^##^: mild-moderate, no oxygen administration (*N* = 73)	35 (47.9)	1.61	0.60–4.31	0.341	1.5	0.42–5.41	0.536
COVID-19 severity^##^: moderate-severe, oxygen administration required (*N* = 46)	37 (80.4)	7.19	2.32–22.35	**0.001**	3.67	0.74–18.19	0.111
Mortality case (*N* = 3)	1 (33.3)	0.39	0.03–4.44	0.451	0.42	0.02–8.10	0.564

*cOR* crude odds ratio, *aOR* adjusted odds ratio, *CI* confidence interval, *var.* variable, *Ref* reference

*P values were calculated by univariate and multivariate logistic regression to compare the cases whose IgG titer >  = 640 and the others

**Adjusted for age, number of days from COVID-19 onset to blood draw, and whether 2nd vaccination given >  = 7 days prior to blood draw

#Missing values were imputed by multiple imputations by chained equation models including all the variables with missing values, IgG titer >  = 640, age, gender, chronic heart disease, diabetes mellitus, dyslipidemia, immunosuppressive agent use, LDH, ALB, BUN, COVID-19 severity, CT scan findings, 2nd vaccination given >  = 7 days prior to blood draw, and whether blood samples were collected for the clinical laboratory tests and IIP on the same day

##COVID-19 severity was defined as follows mild: percutaneous oxygen saturation (SpO2) >  = 96% without pneumonia, mild-moderate: 93% < SpO2 < 96% and/or pneumonia, moderate-severe: SpO2 <  = 93% and oxygen administration request

Of the 146 hospitalized patients, 35 underwent IIP testing on blood samples taken the same day as the admission blood test. Among them, 23 had IgG antibody titers < 640, while 12 had >  = 640. When comparing the laboratory data between the two groups, it was found that in the >  = 640 group, the white blood cell count and hemoglobin A1c were significantly higher, and creatine phosphokinase was significantly lower in univariate analysis. However, after performing multiple imputations for missing values and conducting multivariate analysis, no statistically significant higher (or lower) aOR was observed (Additional file 4).

## Discussion

The findings from the IIP and IgG ELISA tests conducted on COVID-19 inpatients and negative control samples revealed that the IgG antibody testing by IIP produced an ROC curve comparable to that of IgG ELISA. Notably, when the IgG cutoff titer in IIP was >  = 320, the specificity reached 100%, and the sensitivity and accuracy were either equal to or higher than those obtained from ELISA (Additional file 3). One of the reasons for the difference in test results between IIP and ELISA is believed to be the variance in the antigens used. In this study, the ELISA kit utilized a recombinant protein in the S1-domain as an antigen, while the IIP used a whole-cell antigen created by infecting VeroE6/TMPRSS2 cells with SARS-CoV-2. As a result, the ELISA test is particular for the anti-S1 antibody, whereas the IIP is likely to be more sensitive because it detects antibodies to various antigens. In a previous study, the authors compared IgM antibody test results for scrub typhus using a method similar to the IIP test employed in this study with those of an IgM ELISA kit using a recombinant antigen. They noted differences in the test results and concluded that the whole-cell antigen test utilizing an epidemic pathogen is valuable [6]. The SARS-CoV-2 variants prevalent in Fukushima Prefecture during the study period were as follows: R.1 from February to April 2021, Alpha from May to July 2021, Delta from August to December 2021, and Omicron BA.1.1 from January to March 2022. Subsequently, Omicron BA.2 became the predominant variant from April to July 2022, followed by Omicron BA.5 after August 2022, after the study period [11, 12]. Despite using the prevalent variant in this study for IIP testing, the IgG antibody titers were found to be highest against WT-A in almost all samples. To explore this further, the authors requested that the Fukushima Prefectural Institute of Public Health measure the viral load of the antigen cell suspension used for IIP using RT-qPCR. The results showed that the WT-A antigen was 16,636 copies/µL, 40 to 113 times higher than the other variants (PCR protocol: Additional file 1, results: Additional file 3). The findings of the IIP test in this study may have been influenced by the variations in viral load. The susceptibility of cultured cells to SARS-CoV-2 infection may differ based on the variant and cell types used. However, aligning the viral load as closely as possible could provide an advantage when using the prevalent variant. In addition to consistent cell density for each spot in IIP, adjusting the inoculum volume during virus culture and verifying the viral load after inactivating antigenic cells may be essential.

In this study, the IgM test by IIP yielded negative results in 70.5% of COVID-19 patients with a titer of < 40, making it challenging to use as a diagnostic test across different variants and cutoffs during the epidemic. In an IFA antibody test conducted in Australia during the early stages of the epidemic in suspected COVID-19 patients, the sensitivity of IgG was 91.2%, and the sensitivity of IgM was 62.2%. Additionally, the specificity of all tests was more than 99% when using PCR as the reference standard [13]. While the IFA differs from the IIP in its use of fluorescent staining and fluorescent microscope, the principle of the test is similar. The results for COVID-19 are consistent with those of the present study in that the test's sensitivity is significantly different between IgG and IgM. Other findings in a systematic review by the Cochrane COVID-19 Diagnostic Test Accuracy Group indicate that the IgM test by ELISA showed sensitivities of 68.2% (95% CI: 57.1–77.5) and 84.5% (73.5–91.4) in the second and third week from the onset of the disease. RDTs, including LFA, colloidal gold immunoassays, and fluorescence-labeled immunochromatographic assays, demonstrated sensitivities of 63.4% (95% CI: 57.6–68.9) and 76.9% (71.4–81.7) during the same period. Likewise, the IgG test by ELISA showed sensitivities of 63.7% (95% CI: 58.7–68.4) and 89.6% (86.5–92.1), while the RDTs showed sensitivities of 67.6% (95% CI: 63.6–71.5) and 87.1% (84.3–89.4) during the same period [14]. These results suggest a tendency for higher sensitivity in the later phase with the IgG test and ELISA. The IDSA guidelines recommend using IgG or total antibody assays when antibody titers against SARS-CoV-2 are needed. The guidelines neither recommend nor oppose using IgM as a target for the assay. It is also suggested to test for antibodies to the nucleocapsid protein to detect antibody production in COVID-19 in the presence of a history of vaccination, according to the IDSA guidelines [3]. However, identifying specific antigens for antibodies detected by whole-cell antigens using IIP can be challenging. In our study, we could not obtain LFA and other ELISA kits for COVID-19 patients. We anticipate further comparisons between the IIP and other serological tests among COVID-19 patients, particularly focusing on differences in antigen, assay principle, and immunoglobulin subclass.

Of the 36 vaccinated volunteers, ELISA showed the highest frequency of positive IgG antibody tests, with 34 positive results after the first vaccination. IIP had 20 positives, while LFA had 25. Following the second vaccination, both ELISA and IIP showed the same number of positive results, with 35 in each, while LFA had 34 positives. No discrepancies were observed between the IgG test results of ELISA and IIP. Among the 36 volunteers, only one tested positive for IgM with a titer of 80 in IIP after one vaccination, and two tested positive for IgM in LFA. After two vaccinations, four volunteers tested positive for IgM with titers of >  = 80 in IIP, and 12 tested positive in LFA. In summary, the frequency of IgM antibody detection was generally low. All vaccinations given were of the BNT162b2, an mRNA vaccine encoding the full-length spike protein of SARS-CoV-2 [15, 16]. It was observed that following the first vaccination, ELISA and LFA demonstrated a greater detection rate of IgG antibodies compared to IIP. After the second vaccination, LFA exhibited a higher frequency of IgM antibody detection than IIP. This difference is likely attributed to the specific targeting of spike protein antibodies by the ELISA and LFA tests. Additionally, there seemed to be a connection between age and IgG antibody titer after vaccination. Younger participants showed positive IgG antibody titer after the first dose. In contrast, there was a trend toward smaller increases in IgG antibody titer after the second dose for those over 60. However, as this was a small analysis with only 36 volunteers and did not adjust for any factors, it is impossible to definitively claim a true association between age and IgG antibody titer. Nonetheless, the observed negative correlation with age can be considered persuasive evidence of age-related decline in immune response.

A multivariate analysis of factors associated with a significant increase (> = 640) in IgG antibody titers in COVID-19 hospitalized patients revealed several vital associations. These included age, the number of days from the onset of illness to blood draw, the elapsed time since receiving two doses of the vaccine >  = 7 days, and the use of immunosuppressive drugs for comorbidities. Notably, blood drawn more than 14 days after the onset of the disease had the most substantial impact on increasing antibody titers, with an aOR of 37.65. This finding aligns with previous recommendations for IgG testing to be conducted 3–4 weeks after the onset of the disease [3, 4, 14, 17]. Compared to the group aged <  = 50 years, aOR was significantly higher in those aged 50 to 64 at 3.53 but not significant in those aged >  = 65 years. In a previous study, it has been reported that antibody titers tend to increase with age [18]. However, in the present patient group and volunteers, the older age groups showed a smaller increase in antibody titers. The IIP employs whole-cell antigens, which are thought to exhibit a broad range of antigens. It is plausible that middle-aged and older adults have immune memory that aids in increasing antibody titers when infected with SARS-CoV-2, possibly due to previous exposure to non-SARS coronaviruses. Conversely, older individuals are less likely to experience a substantial increase in IgG antibody titers in the IIP compared to middle-aged individuals, even after vaccination. Consequently, the older group is less likely to exhibit as substantial an increase in antibody titers as the middle-aged group. However, it should be noted that this study differs from previous reports in terms of population and race, and unadjusted factors may have influenced the results. The results of the immunosuppressant use should be approached with caution, as only three patients used immunosuppressants in total. Nonetheless, the finding that IgG antibody titers were less likely to be elevated in immunosuppressant users is considered to be compelling. In this study, the relationship between general laboratory tests and antibody titers was only examined in 35 cases, and no significant results were obtained. It would be beneficial to explore the correlation between various biomarkers and antibody titers in a larger sample at different stages of the clinical course, such as at the onset of illness and during the recovery period.

This study has some more limitations. Firstly, it is a single-center study focusing on patients from a specific geographic region eligible for hospitalization. The study site lacked an intensive care unit for COVID-19, making it difficult to include enough severe cases for proper analysis. As a result, the generalizability of the findings may be limited. The potential risk of bias is also caused by the two-gate study design, which tends to overestimate diagnostic parameters. Additionally, it is essential to consider that in the current situation, most patients have received multiple vaccinations and have had SARS-CoV-2 infections. Moreover, there is a wide variety of prevalent variants, so there may be differences between the study's results and the current population trends. Furthermore, the IIP was primarily carried out manually by a single researcher and one laboratory technician. This study represents the first use of the IIP for SARS-CoV-2. Therefore, there may be limitations in validating the test results. Throughout the study, if there were any uncertainties among laboratory personnel regarding the interpretation of the test results, multiple re-tests were conducted to ensure result reproducibility and to reassess the interpretations.

This study illustrates that the performance of the IgG antibody test using the IIP is comparable to that of an ELISA for COVID-19. Several essential points regarding the IIP should be noted. The IIP can be conducted using equipment commonly found in a standard laboratory or research facility without expensive machinery. However, the test does require the provision of inactivated antigen cells. Compared to tests that rely on specialized equipment and costly test kits, such as ELISA, the IIP is considered feasible to conduct in resource-limited settings. Nevertheless, it does require time to become proficient in the manual procedures involved. It is essential to reassess IIP antibody titers in various regions, particularly in areas affected by evolving epidemic variants of SARS-CoV-2 and multiple rounds of vaccinations. Additionally, it is crucial to investigate the implications of high and low IIP antibody titers, including their correlation with protection against infection, prevention of severe disease, risk of over-immunity, and potential complications and long-term effects of COVID-19.

## Conclusions

The diagnostic accuracy of the IIP for detecting IgG antibodies in SARS-CoV-2 infection and after two vaccinations is equivalent to that of an ELISA. In the IIP, IgG antibody titers tended to be higher 14 days or more after disease onset and seven days or more after the second vaccination, although there was a minor increase in the elderly. Further investigations are required to specifically address the association between antibody titers in the IIP and their protective or harmful effects against COVID-19 and post-COVID-19 conditions.

## Supplementary Information

Additional file 1. Additional information for the laboratory methods. The contents of the file are the list of SARS-CoV-2 variants used for IIP, inactivated SARS-CoV-2 infected whole-cell antigen preparation protocol, antigen slide preparation protocol, serum absorption and preparation for IIP test, IIP test protocol, and RT-qPCR protocol for the antigen cell suspensions.

Additional file 2. Picture examples of the IIP antigen cells. a) Negative control, uninfected VeroE6/TMPRSS2 cells spotted on a glass slide, Giemsa staining, seen under × 100 power field. The spotted cells are appropriate in density and distribution. b) Wild type-A variant antigen cells spotted on a glass slide, Giemsa staining, seen under × 100 power field. Several cells show adhesion, ballooning, or syncytium formation as a cytopathic effect due to SARS-CoV-2 virus infection. c) Negative IIP reaction for IgG in 1:40 diluted serum to the negative control cells, seen under × 200 power field. The patient (ID: 27) tested positive for IgG in 1:5120 to wild type-A variant antigen cells. The cells can be faintly observed by reducing the light intensity of the microscope in the negative reaction. d) Positive IIP reaction for IgG in 1:640 diluted serum to wild type-A variant antigen cells, seen under × 200 power field. The patient (ID: 172) tested positive for IgG in 1:10240 to wild type-A variant antigen cells. Apparently, the cell surface where IgG binds to antigens was stained blue-black.

Additional file 3. Additional results of the study. The first section of the file contents is the additional results of IIP and ELISA among COVID-19 patients, including a table and four graphs of days from COVID-19 onset to blood sampling, two tables of the IIP IgM titer among COVID-19 patients with the second vaccination > 7 days before blood sampling and others, and a table of the sensitivity, specificity, and accuracy of the IIP IgG test by different cutoff titer. The second section is the additional results of IIP, ELISA, and LFA among vaccinated volunteers at pre-, post-first, and post-second vaccination, including a graph and table of correlation between the IIP IgG titer and ELISA OD ratio at post-first vaccination, those at post-second vaccination, a graph and table of correlation between the IIP IgG titer and IgM titer at post-second vaccination, two tables of IIP IgG titer by LFA IgG and IgG ELISA results after the first and second vaccination, those of IIP IgM titer, a graph of correlation between log (IIP IgG titer) and days from second vaccination to blood sampling, and a graph of correlation between log (IIP IgG titer) of post-second vaccination and age. The last section presents the additional results of RT-qPCR of antigen cells as a table.

Additional file 4. Additional results of the study participants’ characteristics and laboratory data summary tables with statistical analyses. The file contains tables of the basic characteristics of vaccinated participants, those of COVID-19 patients, COVID-19-related characteristics, and laboratory data of admission patients whose blood samples were collected for the clinical laboratory tests and the IIP on the same day. The additional logistic regression analysis result table for the association between the laboratory data and IgG titer >  = 640 in the IIP is also included.

Additional file 5. Dataset of the study.

## Data Availability

All data generated or analyzed during this study are included in this published article and its supplementary information files.

## Abbreviations

aOR: Adjusted odds ratio
AUC: Area under the receiver operating characteristic curve
ELISA: Enzyme-linked immunosorbent assay
IFA: Immunofluorescent assay
IIP: Indirect immunoperoxidase assay
LFA: Lateral flow assay
OD: Optical density
PBS: Phosphate-buffered saline
RDT: Rapid diagnostic test
ROC: Receiver operating characteristic
RT-PCR: Reverse transcription polymerase chain reaction
WT-A: Wild type-A
