# Supplementary material for: Exploratory study of antibody titers against SARS-CoV-2 using an indirect immunoperoxidase assay in COVID-19 patients and vaccinated volunteers
Source: Trop Med Health. 2024 Sep 29;52:65. doi: 10.1186/s41182-024-00635-y (PMC11439312; doi:10.1186/s41182-024-00635-y)
Supplement: Supplementary file 1 — Additional file 1. Additional information for the laboratory methods. The contents of the file are the list of SARS-CoV-2 variants used for IIP, inactivated SARS-CoV-2 infected whole-cell antigen preparation protocol, antigen slide preparation protocol, serum absorption and preparation for IIP test, IIP test protocol, and RT-qPCR protocol for the antigen cell suspensions. [file 41182_2024_635_MOESM1_ESM.docx]

## List of SARS-CoV-2 variants used for IIP

- hCoV-19/Japan/TY-WK-521/2020 (wild type A) GISAID: EPI_ISL_408667
- hCoV-19/Japan/QHN001/2020 (α variant B.1.1.7) GISAID: EPI_ISL_804007
- hCoV-19/Japan/TY11-927/2021 (δ variant AY.122) GISAID: EPI_ISL_2158617
- hCoV-19/Japan/TY38-871/2021 (ο variant BA.1.1) GISAID: EPI_ISL_7571618
- hCoV-19/Japan/TY40-385/2022 (ο variant BA.2) GISAID: EPI_ISL_9595859
- hCoV-19/Japan/TY41-702/2022 (ο variant BE.1) GISAID: EPI_ISL_13241867

Based on the material transfer agreement, all SARS-CoV-2 variants were provided by the National Institute of Infectious Diseases, Japan to the Fukushima Prefectural Institute of Public Health.

## Inactivated SARS-CoV-2 infected whole-cell antigen preparation protocol

**Note: The following procedures must be performed in the BSL 3 laboratory.**

1. Inoculate VeroE6/TMPRSS2 cells (4.2 x 10^6) in a 75 cm^2^ flask with SARS-CoV-2 (multiplicity of infection 0.5), and culture in DMEM (low glucose) + 10% FBS + 1 mg/mL Geneticin + 100 unit/mL Penicillin + 100 µg/mL Streptomycin under 5% CO_2_ at 37 °C for 14 hours. (VeroE6/TMPRSS2 cell ID: JCRB1819)
2. Discard the liquid medium, wash twice with PBS, and discard the supernatant.
3. After treatment with 0.25% trypsin – 0.02% EDTA, discard the supernatant, and harvest the cells with E-MEM into a 15 mL tube.
4. Centrifuge the tube at 1500 rpm for 5 minutes, and discard the supernatant.
5. Add 4% paraformaldehyde phosphate-buffered solution up to 15 mL and suspend the cells, incubate for >30 minutes at room temperature.
6. Centrifuge the tube at 1500 rpm for 5 minutes, and discard the supernatant.
7. Add PBS up to 15 mL and suspend the cells.
8. Centrifuge the tube at 1500 rpm for 5 minutes, and discard the supernatant.
9. Repeat 7 & 8.
10. Add 1% fetal bovine serum + 0.1% formaldehyde added PBS up to 10 mL and suspend the cells.
11. Store at 4 °C until use, or at -80 °C for storage longer than a couple of days. (The antigen cells may be taken out of the BSL 3 laboratory, as SARS-CoV-2 has already been inactivated.)

For negative control cell preparation, complete the above protocol without inoculation with SARS-CoV-2.

## Antigen slide preparation protocol

1. Dispense 1 mL each of the antigen cell suspension into two 1.5 mL microtubes per antigen.
2. Centrifuge the tubes at 10000 rpm for 5 minutes, and discard the supernatant.
3. Pipet one tube with 1 mL of 0.01 M PBS (pH 7.2) and collect all suspension, then put it into the other tube and mix the same antigen cells by a vortex.
4. Centrifuge the tubes at 500 rpm for 3 minutes, collect 1 mL of the supernatant, then put it into a new 1.5 mL microtube per antigen. (*)
5. Centrifuge the tubes at 10000 rpm for 5 minutes, and discard the supernatant.
6. Add 50 µL each of 0.01 M PBS (pH 7.2) + 0.3% bovine serum albumin and mix well by a vortex.
7. Aspirate the antigen cell suspension by capillary action of a micropipette tip and spot it on a glass slide. (Spots can be put on the same slide with caution to avoid contamination.)
8. Completely dry the slide by air, and stain the spots with Giemsa staining.
9. Check the antigen cell density under a microscope. (Approximately 50 cells/high power field at x400 is recommended.)
10. If the cells are too many, add some 0.01 M PBS (pH 7.2) + 0.3% BSA. If the cells are too few, add 2 – 8 µL of centrifuged sediments (*). Then repeat 7 – 10 until obtaining adequate cell density.
11. Spot antigen cells and negative control cells adjusted for concentration by the above procedure on a glass slide as shown below figure.
12. Dry the slide at 37 °C for 30 minutes, then fix it in acetone at -20 °C for 10 minutes under light-shielded conditions.
13. After drying, use the slide antigen immediately, otherwise keep it in a sealed plastic sachet and store at -20 °C under light-shielded conditions until use. (Caution for use: before opening the seal, place the sachet at room temperature until the slide reaches room temperature to avoid condensation.)


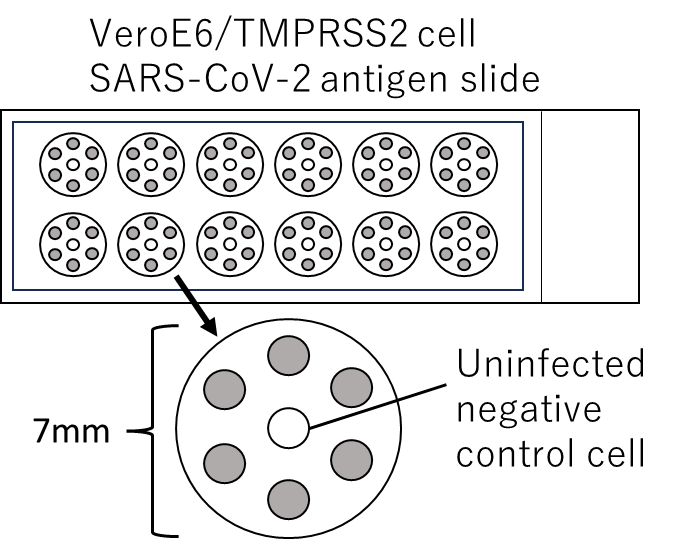


## Serum absorption and preparation for IIP test

1. Thaw stored serum samples and inactivated VeroE6/TEMPRSS2 for negative control (concentration: approximately 200 cells/µL) at room temperature.
2. Dilute the serum 1:10 with 0.01 M PBS (pH 7.2) + 0.3% BSA. (Ex. 50 µL + 450 µL. The diluted samples can be stored at -80 °C until use.)
3. Pipet 20 µL each of the 1:10 diluted serum sample and negative control cells in a new microtube and mix well by a vortex.
4. Incubate the microtube at 37 °C for 30 minutes (1^st^ absorption).
5. Add 40 µL of the negative control cells in the microtube, and incubate the microtube at 37 °C for 30 minutes (2^nd^ absorption).
6. Centrifuge the microtube at 10000 rpm for 5 minutes, and pipet the supernatant in a new tube, which should be tested by IIP as a 1:40 diluted serum sample.

## IIP test protocol

1. Dilute the absorbed serum sample two-fold from 1:40 to 1:10240 with 0.01 M PBS (pH 7.2) + 0.3% BSA. (Ex. Prepare 25 µL of 0.01 M PBS (pH 7.2) + 0.3% BSA in 8 microtubes or 8 wells of a microplate, and pipet 25 µL of a 1:40 diluted serum sample in the 1st one, then dilute sequentially by pipetting.)
2. Carefully load 10 µL of each diluted sample to the spotted antigens on a slide. (Note: avoid touching the antigen slide by the tip of the micropipette.)
3. Incubate the slide in a humidified light-shielded chamber at 37 °C for 30 minutes.
4. Wash the slide with a gentle stream of PBS and rinse in a PBS-filled chamber for 2 minutes, then repeat a 2-minute rinsing with PBS.
5. Carefully load 10 µL of 1:100-diluted horseradish peroxidase-conjugated antihuman IgG or IgM rabbit serum (γ-chain specific or μ-chain specific, Dako Agilent Technologies Japan) to the spotted antigens on a slide. (Note: avoid contamination of the loaded reagents between antigen spots for different dilutions.)
6. Repeat 3 & 4.
7. Incubate the slide in the chamber filled with freshly prepared enzyme substrate solution composed of 1 volume of 80% ethanol containing 0.2% 4-Cl-1-naphtol, 4 volumes of PBS, and 0.01 volume of 3% hydrogen peroxide at room temperature for 5 minutes under light-shielded conditions.
8. Wash the slide three times by changing the solution to distilled water in the chamber. (Pour out the solution, immediately pour in DW, and repeat three times.)
9. After air drying, cover the slide with glycerol gelatin and a coverslip on a warm plate around 30-50 °C.
10. Visually read the results by a light microscope at x100 – x400. (The titer should be determined as the highest dilution of the serum, which demonstrated blue or blue black-colored cellular surface dots.)

## RT-qPCR protocol for the antigen cell suspensions

1. Extract RNA from 140 µL each of the antigen cell suspension following the manufacturer’s instruction using QIAamp Viral RNA Mini (QIAGEN, Cat. No. 52904).
2. Following the manufacturer’s instruction of One Step PrimeScript ^TM^ III RT-qPCR Mix, with UNG (TAKARA BIO INC., Cat. No. RR601A), prepare the reagents mix and set the heat protocol as shown below.

45 cycles

| Reagent | Volume (µL) |
| --- | --- |
| RNase free H_2_O | 3.6 |
| One Step PrimeScript ^TM^ III RT-qPCR Mix, with UNG (2X) | 10.0 |
| # Primer-probe mix (20X):  Forward (400 nM)  Reverse (700 nM)  Probe (200 nM) | 1.0 |
| ROX Reference Dye II (50X) * | 0.4 |
| RNA template | 5.0 |
| Total | 20.0 |

| Temp. | Time |
| --- | --- |
| 25 °C | 10 min. |
| ↓ | |
| 52 °C | 5 min. |
| ↓ | |
| 95 °C | 10 sec. |
| ↓ | |
| 95 °C | 5 sec. |
| ↓↑ | |
| 60 °C | 30 sec. |

*Addition is recommended by the manufacturer for a real-time PCR machine made by Applied Biosystems, etc. (Ex. Applied Biosystems 7500 Real-Time PCR System (Thermo Fisher Scientific))

# NIID-S ver. 2 (S2) set (PCR product size: 149 bp)

| Name | Sequence (5’ to 3’) | Position | Concentration |
| --- | --- | --- | --- |
| SARS-CoV-2_  NIID_S_F1 | CAGTCAGCACCTCATGGTGTA | 24722-24742 | 400 nM |
| SARS-CoV-2_  NIID_S_R3 | AACCAGTGTGTGCCATTTGA | 24870-24851 | 700 nM |
| SARS-CoV-2_  NIID_S_P2 | FAM-TGCTCCTGCCATTTGTCATGATGG  -BHQ1 | 24793-24816 | 200 nM |

Reference: The National Institute of Infectious Diseases, Japan protocol for SARS-CoV-2 gene detection and virus culture Ver. 1.1, 08/02/2021 (URL: <https://www.niid.go.jp/niid/ja/lab-manual-m/10032-sars-cov-ref2.html>), Japanese language.
