# Supplementary figures and images for: Exploratory study of antibody titers against SARS-CoV-2 using an indirect immunoperoxidase assay in COVID-19 patients and vaccinated volunteers
Source: Trop Med Health. 2024 Sep 29;52:65. doi: 10.1186/s41182-024-00635-y (PMC11439312; doi:10.1186/s41182-024-00635-y)

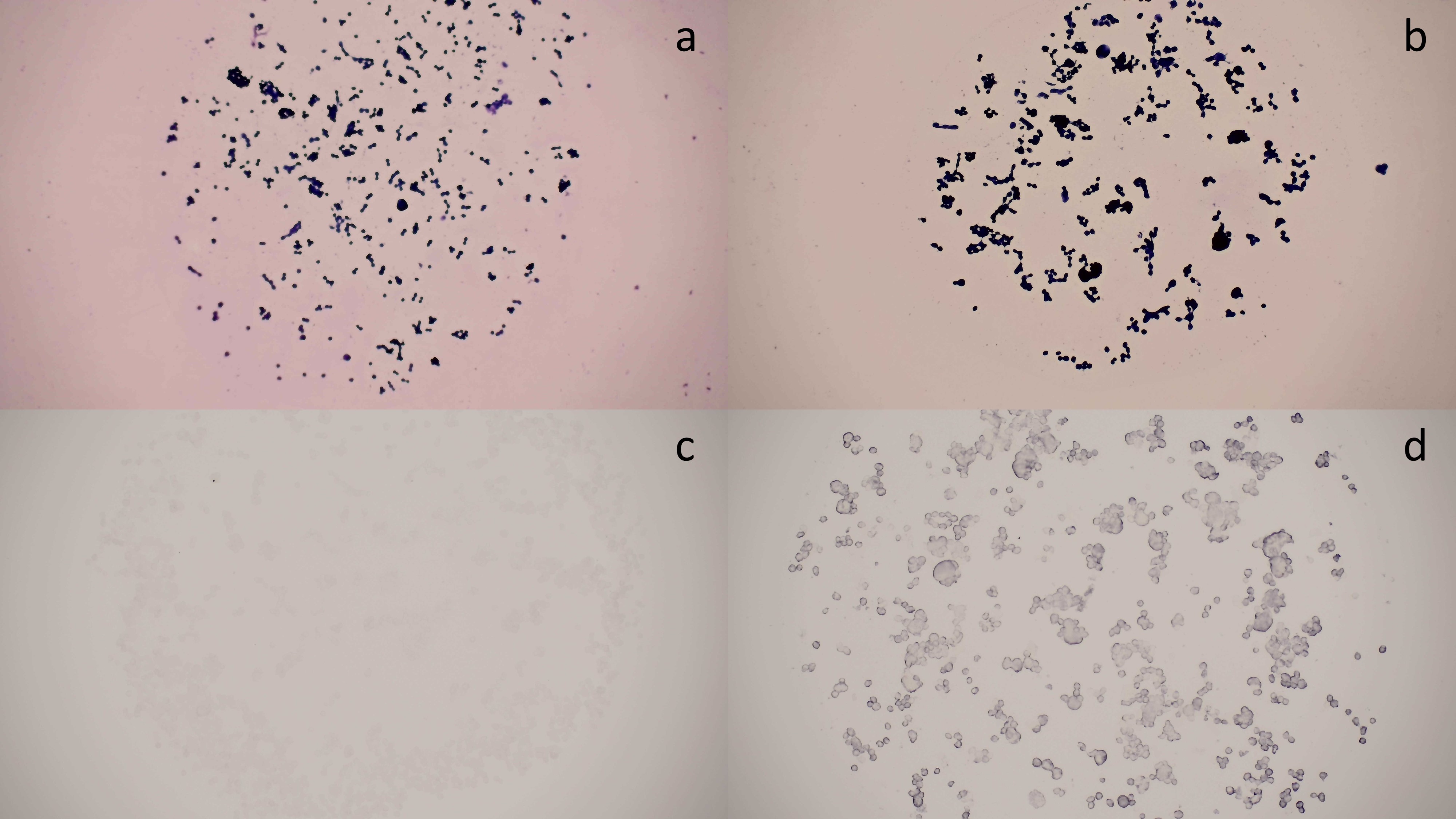

Supplement: Supplementary file 2 — Additional file 2. Picture examples of the IIP antigen cells. a) Negative control, uninfected VeroE6/TMPRSS2 cells spotted on a glass slide, Giemsa staining, seen under × 100 power field. The spotted cells are appropriate in density and distribution. b) Wild type-A variant antigen cells spotted on a glass slide, Giemsa staining, seen under × 100 power field. Several cells show adhesion, ballooning, or syncytium formation as a cytopathic effect due to SARS-CoV-2 virus infection. c) Negative IIP reaction for IgG in 1:40 diluted serum to the negative control cells, seen under × 200 power field. The patient (ID: 27) tested positive for IgG in 1:5120 to wild type-A variant antigen cells. The cells can be faintly observed by reducing the light intensity of the microscope in the negative reaction. d) Positive IIP reaction for IgG in 1:640 diluted serum to wild type-A variant antigen cells, seen under × 200 power field. The patient (ID: 172) tested positive for IgG in 1:10240 to wild type-A variant antigen cells. Apparently, the cell surface where IgG binds to antigens was stained blue-black. [file 41182_2024_635_MOESM2_ESM.jpg]
