# Supplementary material for: Exploratory study of antibody titers against SARS-CoV-2 using an indirect immunoperoxidase assay in COVID-19 patients and vaccinated volunteers
Source: Trop Med Health. 2024 Sep 29;52:65. doi: 10.1186/s41182-024-00635-y (PMC11439312; doi:10.1186/s41182-024-00635-y)
Supplement: Supplementary file 3 — Additional file 3. Additional results of the study. The first section of the file contents is the additional results of IIP and ELISA among COVID-19 patients, including a table and four graphs of days from COVID-19 onset to blood sampling, two tables of the IIP IgM titer among COVID-19 patients with the second vaccination > 7 days before blood sampling and others, and a table of the sensitivity, specificity, and accuracy of the IIP IgG test by different cutoff titer. The second section is the additional results of IIP, ELISA, and LFA among vaccinated volunteers at pre-, post-first, and post-second vaccination, including a graph and table of correlation between the IIP IgG titer and ELISA OD ratio at post-first vaccination, those at post-second vaccination, a graph and table of correlation between the IIP IgG titer and IgM titer at post-second vaccination, two tables of IIP IgG titer by LFA IgG and IgG ELISA results after the first and second vaccination, those of IIP IgM titer, a graph of correlation between log (IIP IgG titer) and days from second vaccination to blood sampling, and a graph of correlation between log (IIP IgG titer) of post-second vaccination and age. The last section presents the additional results of RT-qPCR of antigen cells as a table. [file 41182_2024_635_MOESM3_ESM.docx]

## Additional results of IIP and ELISA among COVID-19 patients

### Days from COVID-19 onset to blood sampling


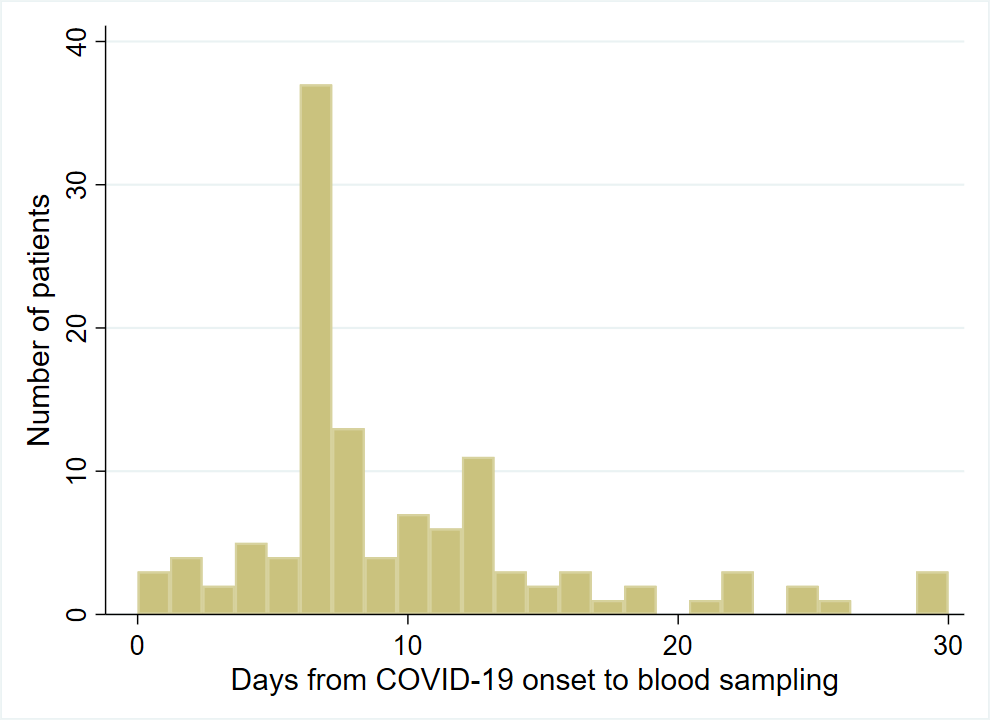


| Days from COVID-19  onset to blood sampling | 0-6 | 7-13 | >=14 | Unknown | Total |
| --- | --- | --- | --- | --- | --- |
| Number of patients | 22 | 74 | 21 | 29 | 146 |


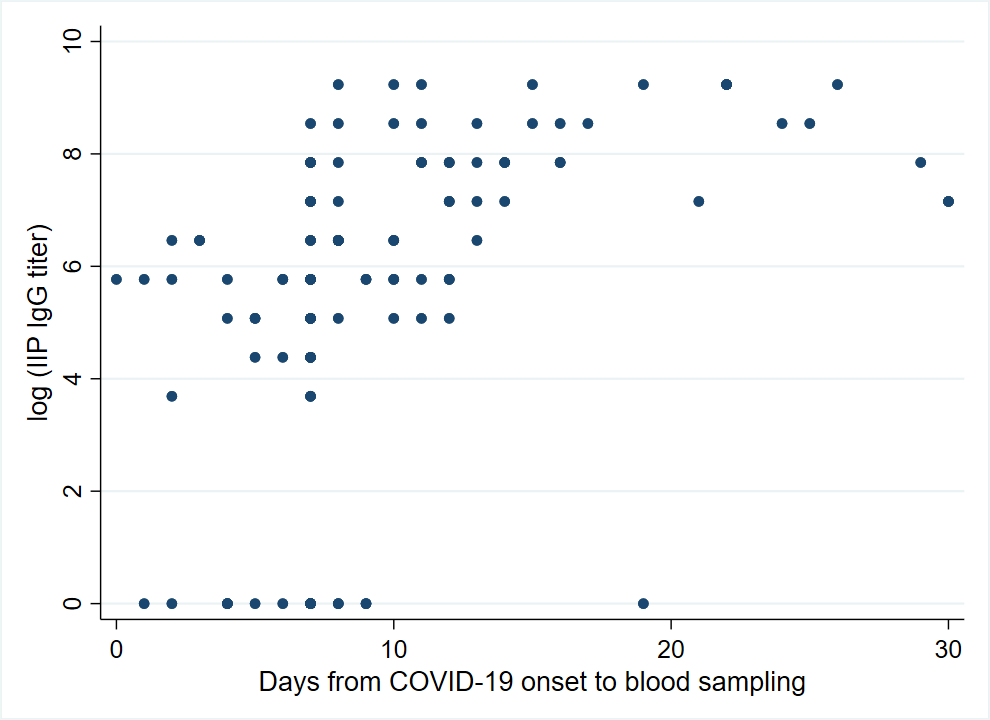

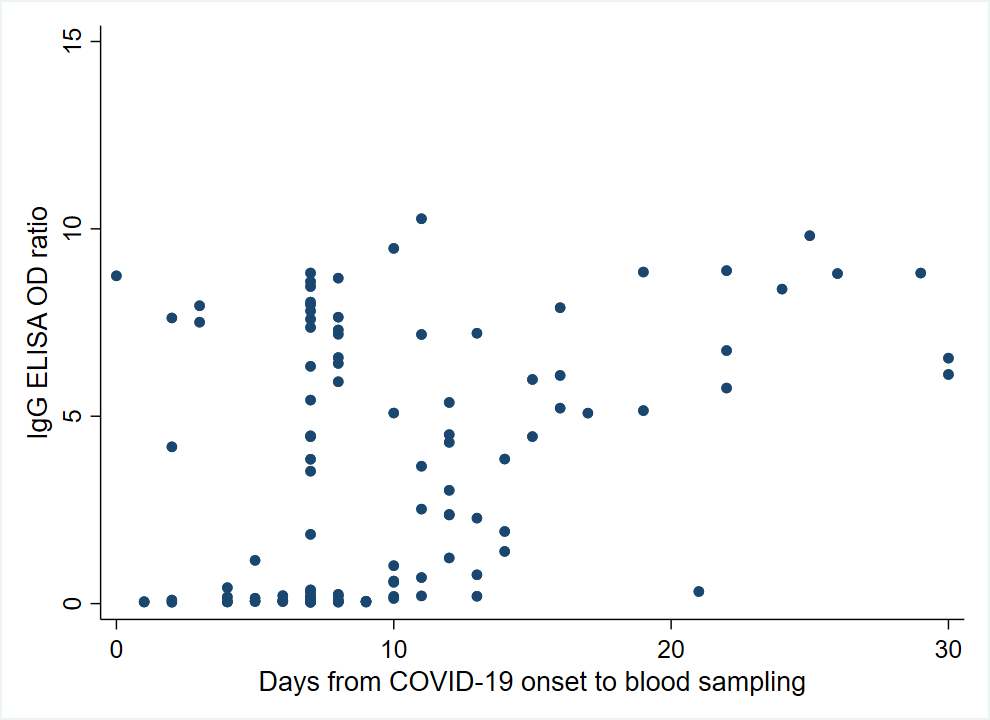


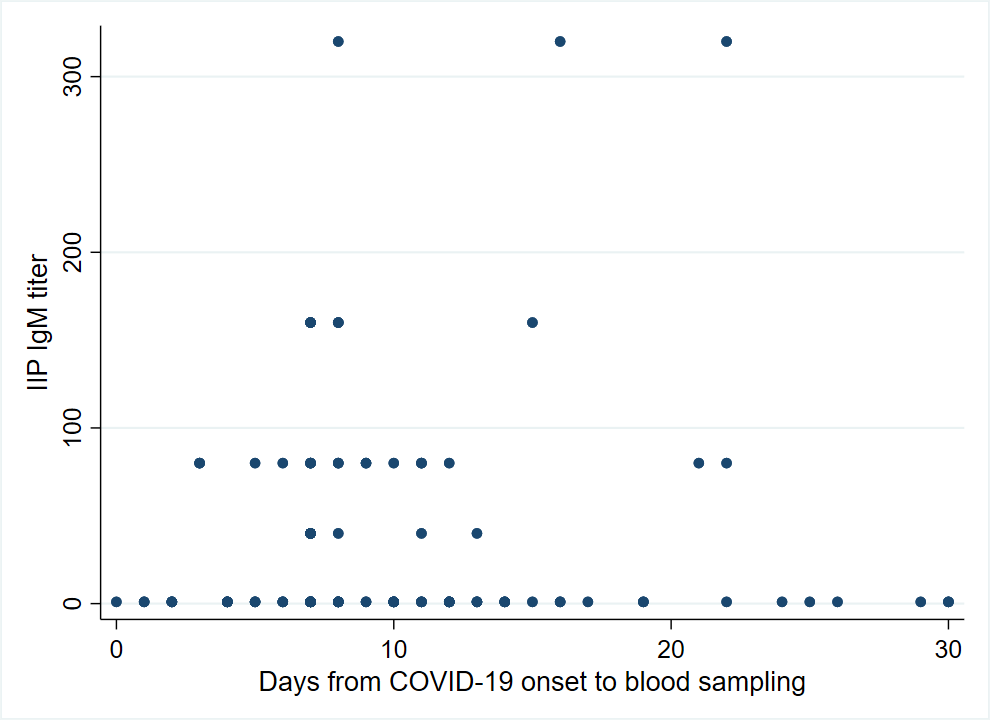


### IIP IgM titer among COVID-19 patients with the second vaccination >7 days before blood sampling and others

| Second vaccination >7 days before blood sampling | |
| --- | --- |
| IIP IgM titer | Number of patients |
| <40 | 10 |
| 40 | 3 |
| 80 | 5 |
| 160 | 5 |
| Total | 23 |

| Others (no vaccination = 117) | |
| --- | --- |
| IIP IgM titer | Number of patients |
| <40 | 93 |
| 40 | 8 |
| 80 | 15 |
| 160 | 4 |
| 320 | 3 |
| Total | 123 |

### Sensitivity, specificity, and accuracy of the IIP IgG test by different cutoff titer

| Cutoff titer | Sensitivity | Specificity | Accuracy |
| --- | --- | --- | --- |
| >=40 | 84.9% | 76.3% | 83.2% |
| >=80 | 82.2% | 94.7% | 84.8% |
| >=160 | 78.1% | 97.4% | 82.1% |
| >=320 | 69.2% | 100% | 75.5% |
| >=640 | 55.5% | 100% | 64.7% |
| >=1280 | 44.5% | 100% | 56.0% |
| >=2560 | 34.3% | 100% | 47.8% |
| >=5120 | 19.2% | 100% | 35.9% |
| >=10240 | 8.9% | 100% | 27.7% |

## Additional results of IIP, ELISA, and LFA among vaccinated volunteers at pre-, post-first, and post-second vaccination.

### Correlation between log (IIP IgG titer) and ELISA OD ratio at post-first vaccination

##
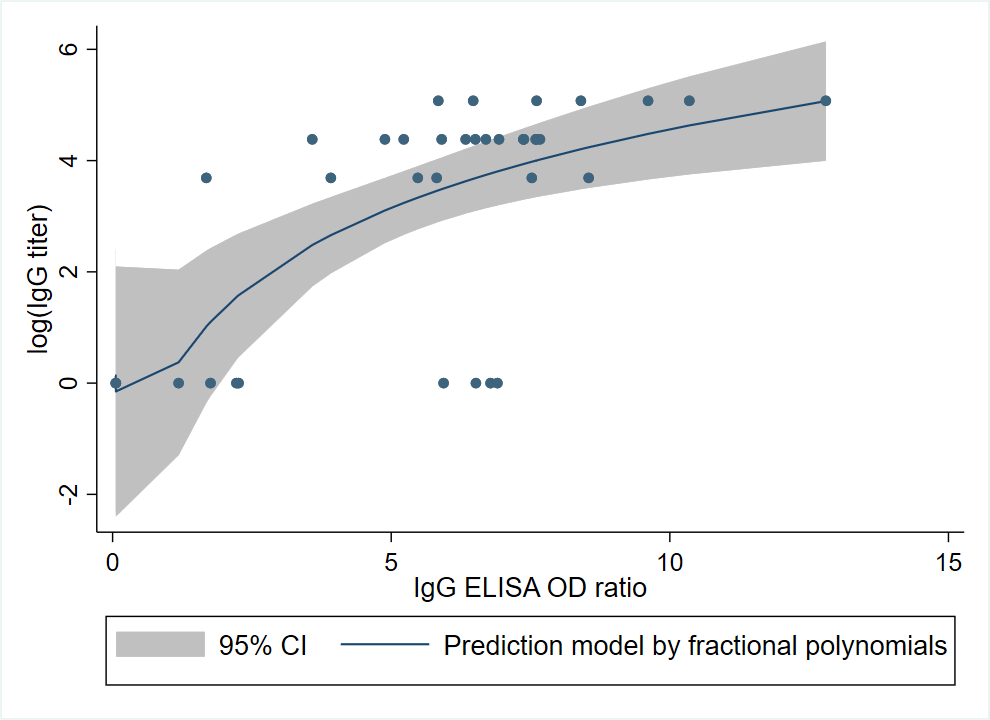


Spearman’s rank correlation: 0.595 (95% CI: 0.332 - 0.773) = moderate correlation

| Post 1^st^ vaccination | | | |
| --- | --- | --- | --- |
| IIP IgG titer | IgG ELISA OD ratio <1.1 | IgG ELISA OD ratio >=1.1 | Total |
| <40 | 2 | 8 | 10 |
| 40 | 0 | 6 | 6 |
| 80 | 0 | 13 | 13 |
| 160 | 0 | 7 | 7 |
| Total | 2 | 34 | 36 |

### Correlation between log (IIP IgG titer) and ELISA OD ratio at post-second vaccination

##
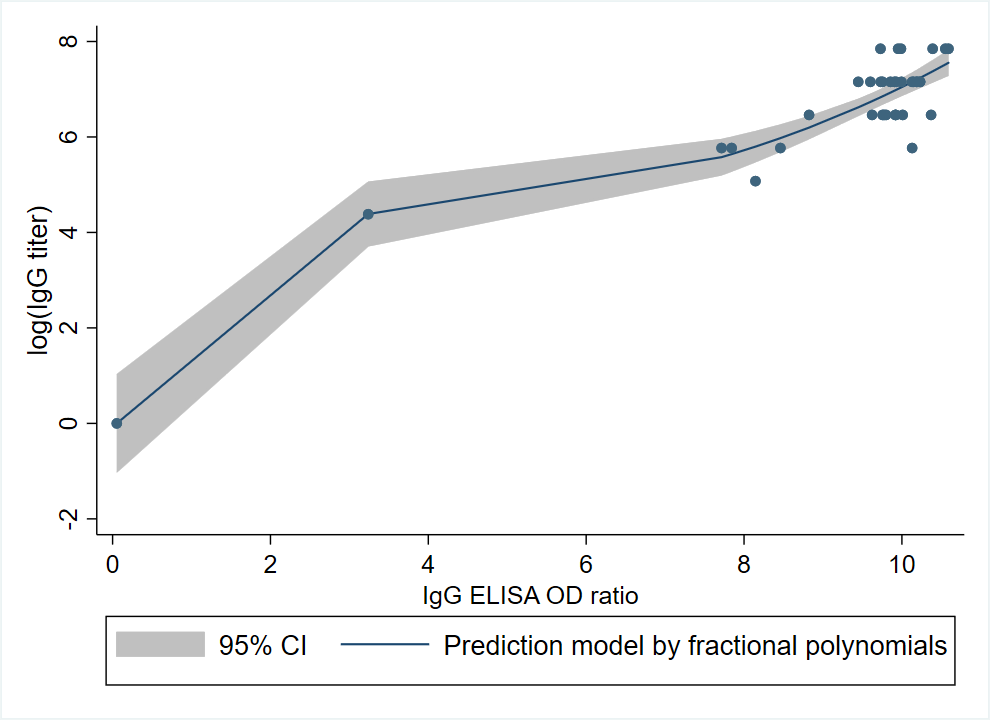


Spearman’s rank correlation: 0.599 (95% CI: 0.337 - 0.775) = moderate correlation

| Post 2^nd^ vaccination | | | |
| --- | --- | --- | --- |
| IIP IgG titer | IgG ELISA OD ratio <1.1 | IgG ELISA OD ratio >=1.1 | Total |
| <40 | 1 | 0 | 1 |
| 80 | 0 | 1 | 1 |
| 160 | 0 | 1 | 1 |
| 320 | 0 | 4 | 4 |
| 640 | 0 | 9 | 9 |
| 1280 | 0 | 14 | 14 |
| 2560 | 0 | 6 | 6 |
| Total | 1 | 35 | 36 |

### Correlation between log (IIP IgG titer) and log (IIP IgM titer) at post-second vaccination

##
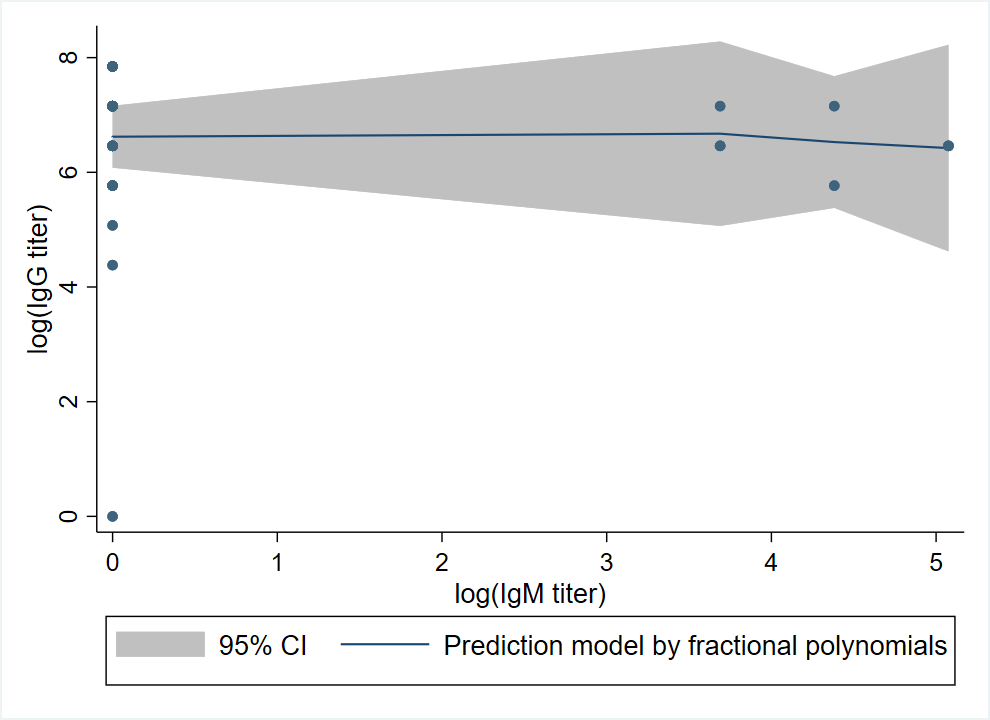


Spearman’s rank correlation: -0.211 (95% CI: -0.504 - 0.125) = no correlation

| Post 2^nd^ vaccination | | | | | |
| --- | --- | --- | --- | --- | --- |
| IIP IgG titer | IIP IgM titer | | | | Total |
|  | <40 | 40 | 80 | 160 |  |
| <40 | 1 | 0 | 0 | 0 | 1 |
| 80 | 1 | 0 | 0 | 0 | 1 |
| 160 | 1 | 0 | 0 | 0 | 1 |
| 320 | 3 | 0 | 1 | 0 | 4 |
| 640 | 5 | 2 | 0 | 2 | 9 |
| 1280 | 12 | 1 | 1 | 0 | 14 |
| 2560 | 6 | 0 | 0 | 0 | 6 |
| Total | 29 | 3 | 2 | 2 | 36 |

### IIP IgG titer by LFA IgG and IgG ELISA results

| Post 1^st^ vaccination | | | |
| --- | --- | --- | --- |
| IIP IgG titer | LFA IgG  (-) | LFA IgG  (+) | Total |
| <40 | 6* | 4 | 10* |
| 40 | 2 | 4 | 6 |
| 80 | 3 | 10 | 13 |
| 160 | 0 | 7** | 7** |
| Total | 11* | 25** | 36*^,^ ** |

* Two samples tested negative, and the other 34 samples tested positive by IgG ELISA.

** Two samples tested by x2 dilution by LFA due to insufficient sample volume.

| Post 2^nd^ vaccination | | | |
| --- | --- | --- | --- |
| IIP IgG titer | LFA IgG  (-) | LFA IgG  (+) | Total |
| <40 | 1* | 0 | 1* |
| 80 | 1 | 0 | 1 |
| 160 | 0 | 1 | 1 |
| 320 | 0 | 4 | 4 |
| 640 | 0 | 9 | 9 |
| 1280 | 0 | 14 | 14 |
| 2560 | 0 | 6 | 6 |
| Total | 2* | 34 | 36* |

* One sample tested negative, and the other 35 samples tested positive by IgG ELISA.

### IIP IgM titer by LFA IgM and IgG ELISA results

| Post 1^st^ vaccination | | | |
| --- | --- | --- | --- |
| IIP IgM titer | LFA IgM  (-) | LFA IgM  (+) | Total |
| <40 | 33*^,^ ** | 2 | 35*^,^ ** |
| 80 | 1 | 0 | 1 |
| Total | 34*^,^ ** | 2 | 36*^,^ ** |

* Two samples tested negative, and the other 34 samples tested positive by IgG ELISA.

** Two samples tested by x2 dilution by LFA due to insufficient sample volume.

| Post 2^nd^ vaccination | | | |
| --- | --- | --- | --- |
| IIP IgM titer | LFA IgM  (-) | LFA IgM  (+) | Total |
| <40 | 19* | 10 | 29* |
| 40 | 1 | 2 | 3 |
| 80 | 2 | 0 | 2 |
| 160 | 2 | 0 | 2 |
| Total | 24* | 12 | 36* |

* One sample tested negative, and the other 35 samples tested positive by IgG ELISA.

### Correlation between log (IIP IgG titer) and days from second vaccination to blood sampling

##
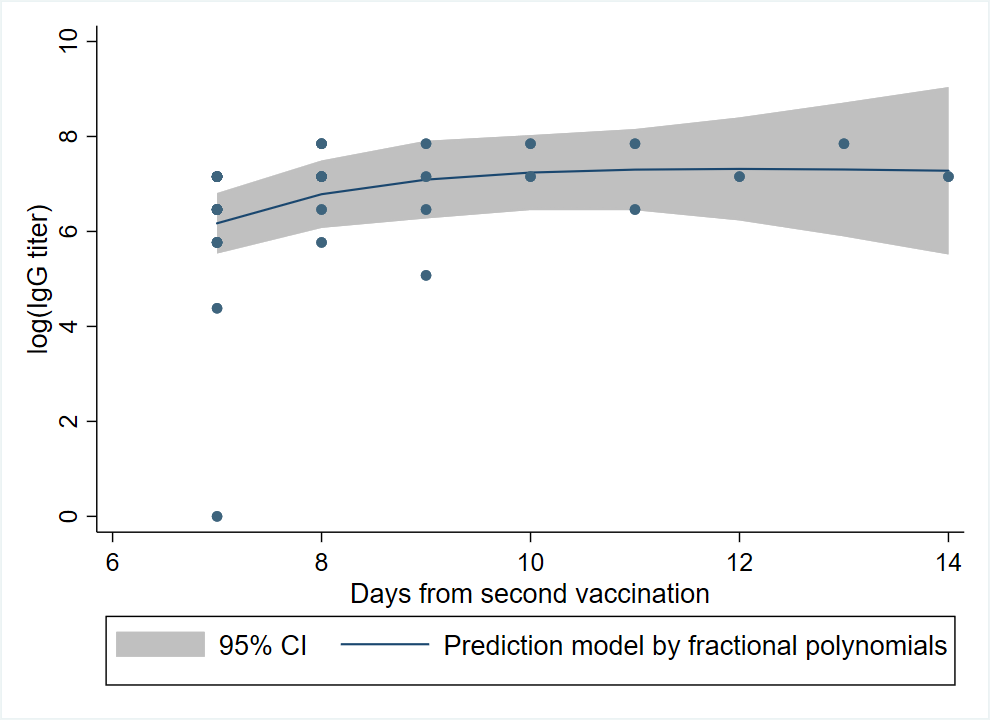


Spearman’s rank correlation: 0.443 (95% CI: 0.134 - 0.673) = moderate correlation

IgG titer seems at plateau after 10th day.

### Correlation between log (IIP IgG titer) of post-second vaccination and age

##
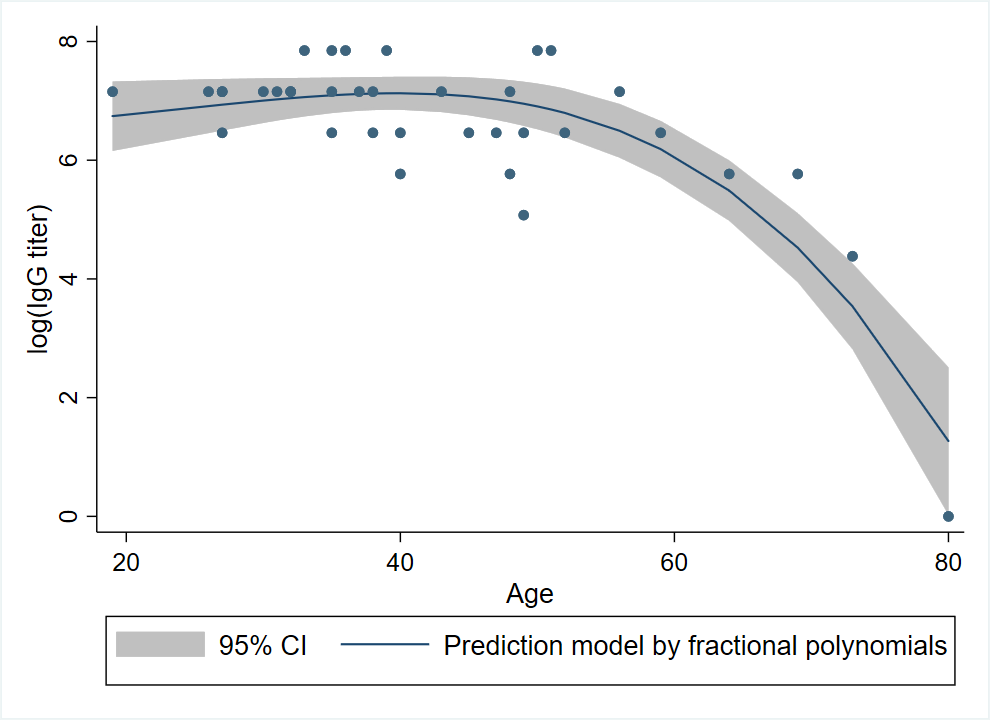


Spearman’s rank correlation: -0.483 (95% CI: -0.700 - -0.183) = moderate correlation

IgG titer seems lower among age >60.

## Additional results of RT-qPCR of antigen cells

| Sample Name | Ct Mean | Copies/µL |
| --- | --- | --- |
| WK (WT-A) | 26.0 | 16,636 |
| QHN (α) | 31.7 | 409 |
| TY11 (δ) | 31.9 | 351 |
| TY38 (ο-BA.1.1) | 33.2 | 147 |
| TY40 (ο-BA.2) | 31.6 | 419 |
| TY41 (ο-BA.5) | 32.1 | 314 |
| *20hr-TY38 (ο-BA.1.1) | 32.1 | 314 |
| *20hr-TY40 (ο-BA.2) | 30.7 | 784 |
| *20hr-TY41 (ο-BA.5) | 31.8 | 381 |
| NC (VeroE6/TMPRSS2) | (-) | (-) |

* Cells were cultured for 20 hours (instead of 14 hours) after SARS-CoV-2 infection.
