# Supplementary material for: Exploratory study of antibody titers against SARS-CoV-2 using an indirect immunoperoxidase assay in COVID-19 patients and vaccinated volunteers
Source: Trop Med Health. 2024 Sep 29;52:65. doi: 10.1186/s41182-024-00635-y (PMC11439312; doi:10.1186/s41182-024-00635-y)
Supplement: Supplementary file 4 — Additional file 4. Additional results of the study participants’ characteristics and laboratory data summary tables with statistical analyses. The file contains tables of the basic characteristics of vaccinated participants, those of COVID-19 patients, COVID-19-related characteristics, and laboratory data of admission patients whose blood samples were collected for the clinical laboratory tests and the IIP on the same day. The additional logistic regression analysis result table for the association between the laboratory data and IgG titer > = 640 in the IIP is also included. [file 41182_2024_635_MOESM4_ESM.pdf]

Basic characteristics of vaccinated participants	Vaccinated volunteer total N=36 n (%)	Post 1st vac. IIP_IgG titer <80 N=16 n (%)	Post 1st vac. IIP_IgG titer >=80 N=20 n (%)	P value*
Age (mean, SD), years	42.7, 13.9	48.6, 16.2	38.2, 10.0	**0.024**
Age <50	27 (75.0)	10 (62.5)	17 (85.0)	0.13
Age 50-64	6 (16.7)	3 (18.8)	3 (15.0)	
Age >=65	3 (8.3)	3 (18.8)	0 (0.0)	
Male sex	15 (41.7)	9 (56.3)	6 (30.0)	0.175
Chronic heart disease	0 (0.0)	0 (0.0)	0 (0.0)	NA
Chronic lung disease	2 (5.6)	1 (6.3)	1 (5)	1
Autoimmune disease	0 (0.0)	0 (0.0)	0 (0.0)	NA
Cirrhosis	0 (0.0)	0 (0.0)	0 (0.0)	NA
Malignant disease	4 (11.1)	1 (6.3)	3 (15.0)	0.613
Chronic kidney disease	0 (0.0)	0 (0.0)	0 (0.0)	NA
Hemodialysis	0 (0.0)	0 (0.0)	0 (0.0)	NA
Diabetes mellitus	2 (5.6)	2 (12.5)	0 (0.0)	0.19
Hypertension	7 (19.4)	5 (31.3)	2 (10.0)	0.204
Dyslipidemia	3 (8.3)	3 (18.8)	0 (0.0)	0.078
Body mass index >=30	0 (0.0)	0 (0.0)	0 (0.0)	NA
Pregnancy	0 (0.0)	0 (0.0)	0 (0.0)	NA
Steroid use for comorbidity	1 (2.8)	1 (6.3)	0 (0.0)	0.444
Immunosuppressive agent for comorbidity	1 (2.8)	1 (6.3)	0 (0.0)	0.444
Current smoker	9 (25.0)	6 (37.5)	3 (15.0)	0.146
Ex-smoker	20 (55.6)	10 (62.5)	10 (50.0)	0.515
Past history of COVID-19	0 (0.0)	0 (0.0)	0 (0.0)	NA

* P values were calculated by Fisher's exact test for categorical variables, Student's t-test for continuous variables.
vac.: vaccinated, IIP_IgG: indirect immunoperoxidase test for immunoglobulin G, SD: standard deviation, NA: not applicable

Basic characteristics of COVID-19 patients	Admission cases total N=146 n (%)	IIP_IgG titer <640 N=65 n (%)	IIP_IgG titer >=640 N=81 n (%)	P value*
Age (mean, SD), years	55.9, 20.1	49.5, 20.5	61.1, 18.4	**<0.001**
Age <50	57 (39.0)	36 (55.4)	21 (25.9)	
Age 50-64	40 (27.4)	12 (18.5)	28 (34.6)	**0.001**
Age >=65	49 (33.6)	17 (26.2)	32 (39.5)	
Male sex	76 (52.1)	34 (52.3)	42 (51.9)	1
Chronic heart disease	14 (9.6)	7 (10.8)	7 (8.6)	0.78
Chronic lung disease	5 (3.4)	2 (3.1)	3 (3.7)	1
Autoimmune disease	4 (2.7)	2 (3.1)	2 (2.5)	1
Cirrhosis	0 (0)	0 (0)	0 (0)	NA
Malignant disease	6 (4.1)	3 (4.6)	3 (3.7)	1
Chronic kidney disease	4 (2.7)	1 (1.5)	3 (3.7)	0.629
Hemodialysis	0 (0)	0 (0)	0 (0)	NA
Diabetes mellitus	33 (22.6)	10 (15.4)	23 (28.4)	0.074
Hypertension	60 (41.1)	18 (27.7)	42 (51.9)	**0.004**
Dyslipidemia	33 (22.6)	8 (12.3)	25 (30.9)	**0.009**
Body mass index >=30	8 (5.5)	3 (4.6)	5 (6.2)	0.733
Pregnancy	0 (0)	0 (0)	0 (0)	NA
Steroid use for comorbidity	6 (4.1)	2 (3.1)	4 (4.9)	0.693
Immunosuppressive agent for comorbidity	3 (2.1)	1 (1.5)	2 (2.5)	1
Current smoker	28 (20.3) (N=138)	15 (23.8) (N=63)	13 (17.3) (N=75)	0.399
Ex-smoker	63 (45.7) (N=138)	32 (50.8) (N=63)	31 (41.3) (N=75)	0.305
Past history of COVID-19	0 (0)	0 (0)	0 (0)	NA

* P values were calculated by Fisher's exact test for categorical variables, Student's t-test for normally distributed continuous variables, and Wilcoxon's rank-sum test for other continuous variables.

IIP_IgG: indirect immunoperoxidase test for immunoglobulin G, SD: standard deviation, NA: not applicable

COVID−19 related characteristics	Admission cases total N=146 n (%)	IIP_IgG titer <640 N=65 n (%)	IIP_IgG titer >=640 N=81 n (%)	P value*
Days from COVID-19 onset to blood draw (mean, SD)	9.8, 6.0 (N=117)	6.9, 3.1 (N=59)	12.8, 6.8 (N=58)	**<0.001**
Days from COVID-19 onset to blood draw: <7 days	22 (18.8)	19 (32.2)	3 (5.2)	
Days from COVID-19 onset to blood draw: 7-13 days	74 (63.3)	39 (66.1)	35 (60.3)	**<0.001**
Days from COVID-19 onset to blood draw: >=14 days	21 (17.9)	1 (1.7)	20 (34.5)	
Days from 2nd vaccination to blood draw (median, IQR)	110.5, 47-182 (N=20)	80, 36-176 (N=7)	140, 63-188 (N=13)	0.475
Days from 2nd vaccination to blood draw: >=7 days	23 (15.8)	7 (10.8)	16 (19.8)	0.173
CT scan finding of COVID-19 associated pneumonia: none	29 (19.9)	17 (26.2)	12 (14.8)	
CT scan finding of COVID-19 associated pneumonia: in 1 lobe	21 (14.4)	14 (21.5)	7 (8.6)	**0.008**
CT scan finding of COVID-19 associated pneumonia: in >=2 lobes	96 (65.8)	34 (52.3)	62 (76.5)	
Any COVID-19 symptom	144 (98.6)	64 (98.5)	80 (98.8)	1
COVID-19 severity**: asymptomatic	2 (1.4)	1 (1.5)	1 (1.2)	
COVID-19 severity**: mild	22 (15.1)	14 (21.5)	8 (9.9)	
COVID-19 severity**: mild-moderate, oxygen administration not required	73 (50)	38 (58.5)	35 (43.2)	**<0.001**
COVID-19 severity**: moderate-severe, oxygen administration required	46 (31.5)	9 (13.9)	37 (45.7)	
COVID-19 severity**: severe	3 (2.1)	3 (4.6)	0 (0)	
Mortality case	3 (2.1)	2 (3.1)	1 (1.2)	0.585

* P values were calculated by Fisher's exact test for categorical variables, Student's t-test for normally distributed continuous variables, and Wilcoxon’s rank-sum test for other continuous variables.
** COVID-19 severity was defined as follows, mild: percutaneous oxygen saturation(SpO2) >=96% without pneumonia, mild-moderate: 93%<SpO2<96% and/or pneumonia, moderate-severe: SpO2 <=93% and oxygen administration request, severe: intensive care and/or mechanical ventilation request.
IIP_IgG: indirect immunoperoxidase test for immunoglobulin G, SD: standard deviation, IQR: interquartile range

Laboratory data**	Admission cases total N=35 (median, IQR)	IIP_IgG titer <640 N=23 (median, IQR)	IIP_IgG titer >=640 N=12 (median, IQR)	P value*
WBC (x1000/μL)	5.29, 4.72-7.5	4.99, 4.66-6.54	7.23, 5.22-7.87	**0.042**
Neutrophil percentage (%)	69, 61.8-79.2	67.5, 60.5-79.2	70.6, 62.7-78.9	0.404
Lymphocyte percentage (%)	20.2, 13.5-27.7	20, 13.1-28.7	21.2, 13.7-26.8	0.862
Platelet (x1000/μL)	209, 176-246	199, 162-236	226, 188-270	0.126
D-dimer (μg/mL)	0.25, 0.25-0.9	0.25, 0.25-0.6	0.43, 0.25-1.05	0.407
Total bilirubin (mg/dL)	0.49, 0.35-0.72	0.48, 0.36-0.72	0.51, 0.34-0.66	0.889
AST (IU/L)	25, 18-31	25, 18-33	22, 15.5-28	0.153
ALT (IU/L)	21, 14-34	21, 14-32	20.5, 15.5-35.5	1
LDH (IU/L)	189, 153-223	181, 151-245	198.5, 158-211	0.715
CPK (IU/L)	54, 44-104	72, 47-144	49.5, 26.5-60.5	**0.026**
CRP (mg/dL)	0.45, 0.08-1.91	0.45, 0.08-2.23	0.16, 0.06-1.84	0.664
Alubumin (g/dL)	4.3, 3.8-4.7	4.5, 4-4.8	3.9, 3.45-4.55	0.121
BUN (mg/dL)	13, 9.3-17.8	11.8, 9.3-17.1	13.8, 10.5-17.8	0.702
Creatinine (mg/dL)	0.71, 0.59-0.83	0.71, 0.55-0.83	0.71, 0.66-0.84	0.52
HbA1c (%)	6, 5.5-6.7	5.7, 5.5-6.2	6.35, 5.85-7.3	**0.018**

* P values were calculated by Wilcoxon's rank-sum test for not normally distributed continuous variables.

** Laboratory data analysis was conducted for admission cases whose blood samples were collected for the clinical laboratory tests and IIP_IgG on the same day.

IIP_IgG: indirect immunoperoxidase test for immunoglobulin G, IQR: interquartile range, WBC: white blood cell, AST: aspartate transaminase, ALT: alanine transaminase, LDH: lactate dehydrogenase, CPK: creatine phosphokinase, CRP: c-reactive protein, BUN: blood urea nitrogen, HbA1c: hemoglobin A1c

Logistic regression analysis for association with IIP_IgG titer >=640 in admission cases n=12 (total observation N=35)

Laboratory data[#]	IgG titer >=640, n (%)	cOR	95%CI	P value*	aOR**	95%CI	P value*
WBC as a continuous var.	12 (34.3)	1	1.00-1.00	0.383	1	1.00-1.00	0.82
Neutrophil percentage as a continuous var.	12 (34.3)	1.02	0.96-1.09	0.518	0.86	0.74-1.02	0.076
Lymphocyte percentage as a continuous var.	12 (34.3)	0.98	0.91-1.07	0.709	1.16	0.98-1.36	0.086
Platelet as a continuous var.	12 (34.3)	1	1.00-1.00	0.115	1	1.00-1.00	0.083
D-dimer as a continuous var.	12 (34.3)	1.21	0.82-1.78	0.341	0.46	0.15-1.45	0.186
Total bilirubin as a continuous var.	12 (34.3)	0.56	0.07-4.24	0.576	0.45	0.04-5.72	0.537
AST as a continuous var.	12 (34.3)	0.94	0.86-1.02	0.153	0.97	0.88-1.06	0.467
ALT as a continuous var.	12 (34.3)	1	0.96-1.04	0.808	1	0.95-1.05	0.925
LDH as a continuous var.	12 (34.3)	0.99	0.98-1.01	0.315	0.99	0.97-1.01	0.328
CPK as a continuous var.	12 (34.3)	0.97	0.94-1.00	0.055	0.94	0.86-1.02	0.113
CRP as a continuous var.	12 (34.3)	1.07	0.78-1.45	0.685	1.08	0.72-1.61	0.714
Alubumin as a continuous var.	12 (34.3)	0.4	0.13-1.27	0.119	1.58	0.07-33.88	0.771
BUN as a continuous var.	12 (34.3)	1	0.92-1.08	0.963	0.86	0.69-1.06	0.16
Creatinine as a continuous var.	12 (34.3)	1.91	0.04-93.71	0.744	4.9	0.01-3351.7	0.633
HbA1c as a continuous var. (N=33)[##]	12 (36.4)	2.44	0.97-6.16	0.058	7.5[###]	0.41-138.4	0.174

* P values were calculated by univariate and multivariate logistic regression to compare the cases whose IIP_IgG titer >=640 and the others.
** Adjusted for age, number of days from COVID-19 onset to blood draw, and whether 2nd vaccination given >=7days prior to blood draw.
# Laboratory data analysis was conducted for patients whose blood samples were collected for the clinical tests and IIP_IgG on the same day.
Missing values were imputed by multiple imputations by chained equation models including all the variables with missing values, IIP_IgG titer >=640, age, gender, chronic heart disease, diabetes mellitus, dyslipidemia, immunosuppressive agent use, LDH, ALB, BUN, COVID-19 severity, CT scan findings, 2nd vaccination given >=7days prior to blood draw, and whether blood samples were collected for the clinical laboratory tests and IIP_IgG on the same day.
Age was excluded from the multivariate model, because of failure in convergence of log likelihood due to the small sample size and skewed data distribution.
IIP_IgG: indirect immunoperoxidase test for immunoglobulin G, cOR: crude odds ratio, aOR: adjusted odds ratio, CI: confidence interval, var.: variable, WBC: white blood cell, AST: aspartate transaminase, ALT: alanine transaminase, LDH: lactate dehydrogenase, CPK: creatine phosphokinase, CRP: c-reactive protein, BUN: blood urea nitrogen, HbA1c: hemoglobin A1c
